# Supplementary material for: A Study on the Direct Esterification of Monoalkylphosphates and Dialkylphosphates; The Conversion of the Latter Species to Trialkylphosphates by Alkylating Esterification
Source: Molecules. 2022 Jul 22;27(15):4674. doi: 10.3390/molecules27154674 (PMC9331942; doi:10.3390/molecules27154674)
Supplement: Supplementary file 1 [file molecules-27-04674-s001.zip › molecules-1827834-supplementary.pdf]

# Supporting Information

## A Theoretical Study on the Direct Esterification of Monoalkylphosphates and Dialkylphosphates; The Conversion of the Latter Species to Trialkylphosphates by Alkylating Esterification

Péter Ábrányi-Balogh, Nikoletta Harsági, László Drahos and György Keglevich *

## Content

**$^{31}$P, $^{13}$C and $^{1}$H NMR spectra for the products (in $CDCl_3$)** ........ 2
*Dibutyl-ethylphosphate (7a)* ........ 2
*Dibutyl-propylphosphate (7b)* ........ 4
*Dibutyl-isopropylphosphate (7c)* ........ 6
*Dibutyl-pentylphosphate (7d)* ........ 8
*Dipentyl-ethylphosphate (8a)* ........ 10
*Dipentyl-propylphosphate (8b)* ........ 12
*Dipentyl-isopropylphosphate (8c)* ........ 14
*Dipentyl-butylphosphate (8d)* ........ 16
**Computed raw data** ........ 18
**Calculated data** ........ 18
**XYZ geometries of computed species** ........ 19

# $^{31}P$, $^{13}C$ and $^{1}H$ NMR spectra for the products (in $CDCl_3$)

***Dibutyl-ethylphosphate (7a)***

$^{31}P$ NMR (202.4 MHz, $CDCl_3$)

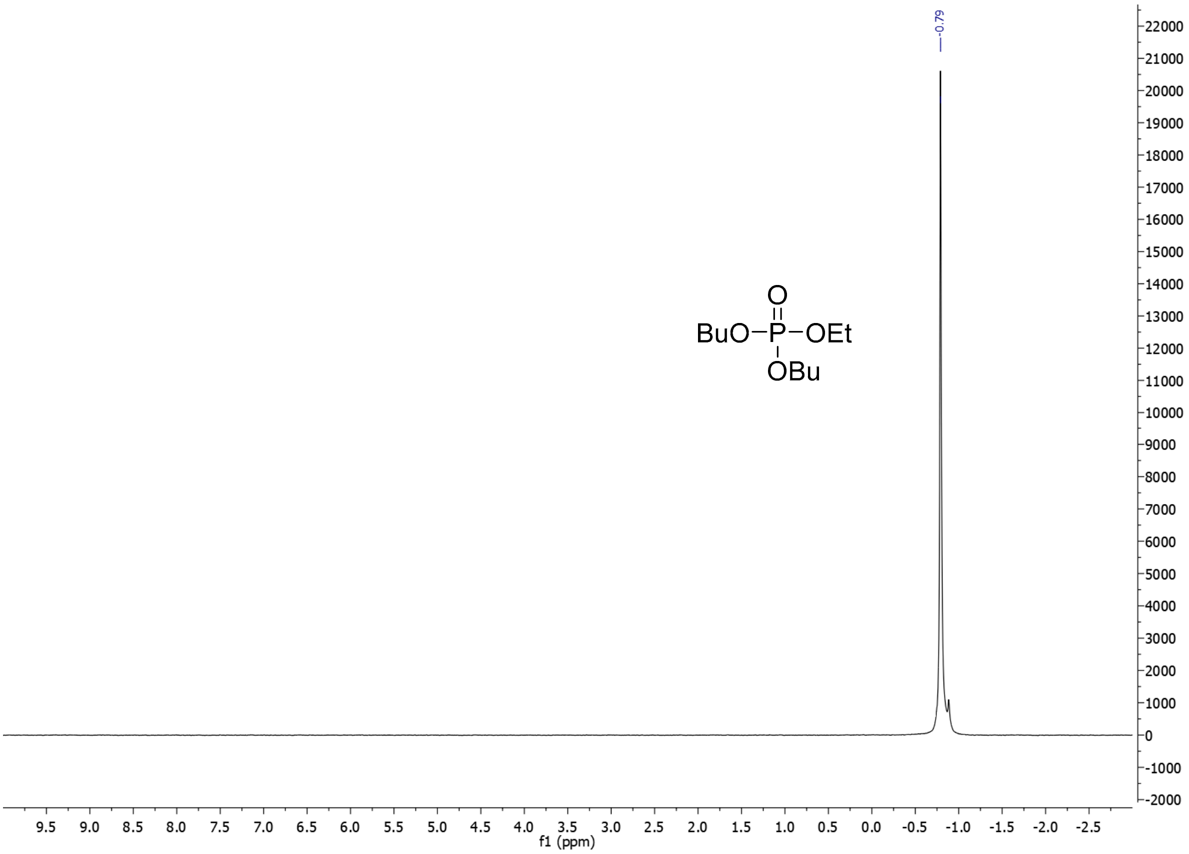

$^{13}C$ NMR (125.7 MHz, $CDCl_3$)

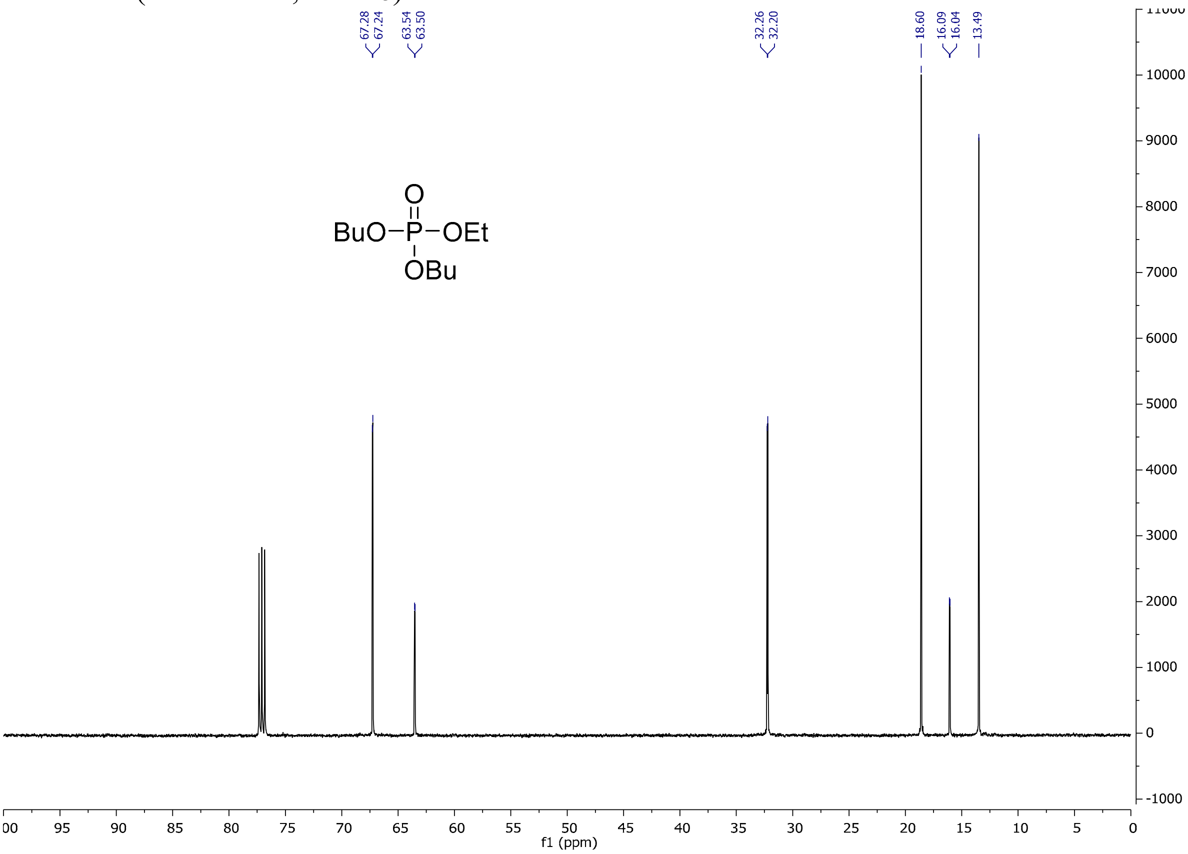


$^{1}H$ NMR (500 MHz, $CDCl_3$)

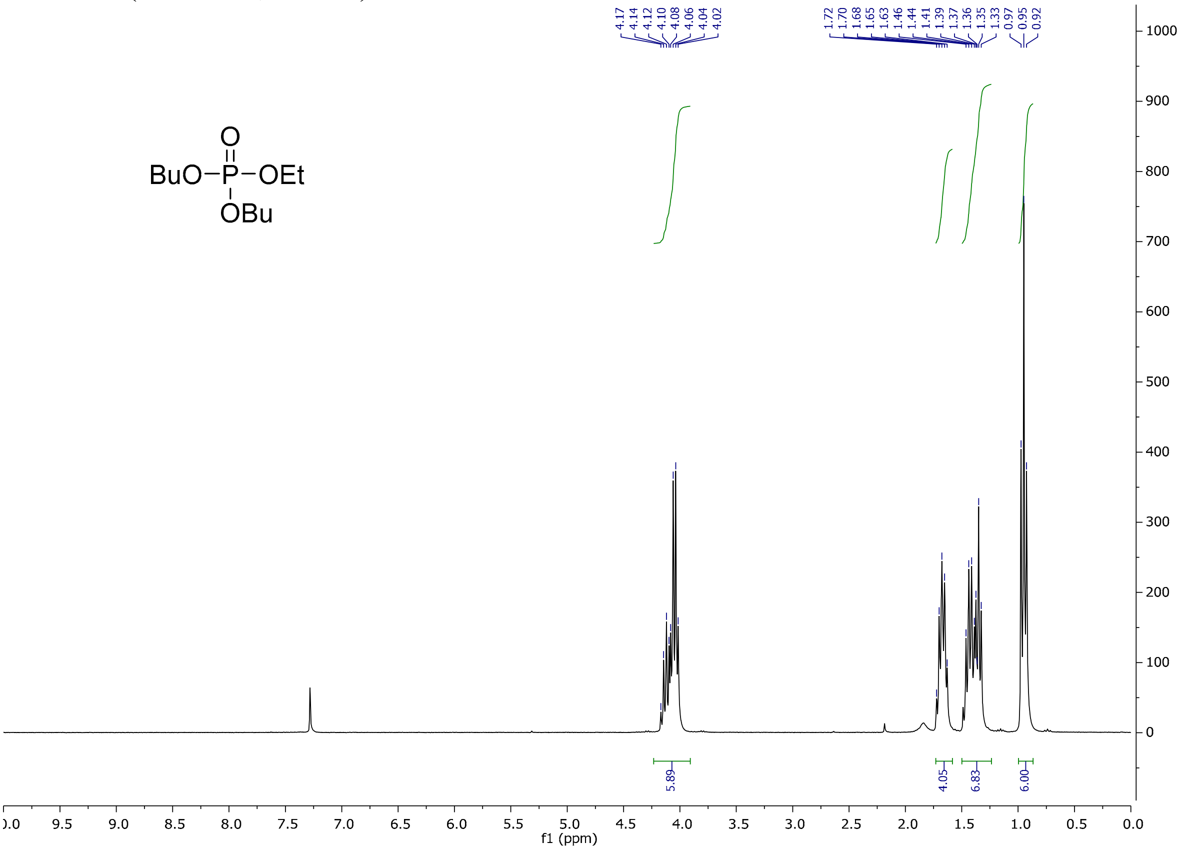

***Dibutyl-propylphosphate (7b)***

$^{31}P$ NMR (202.4 MHz, $CDCl_3$)

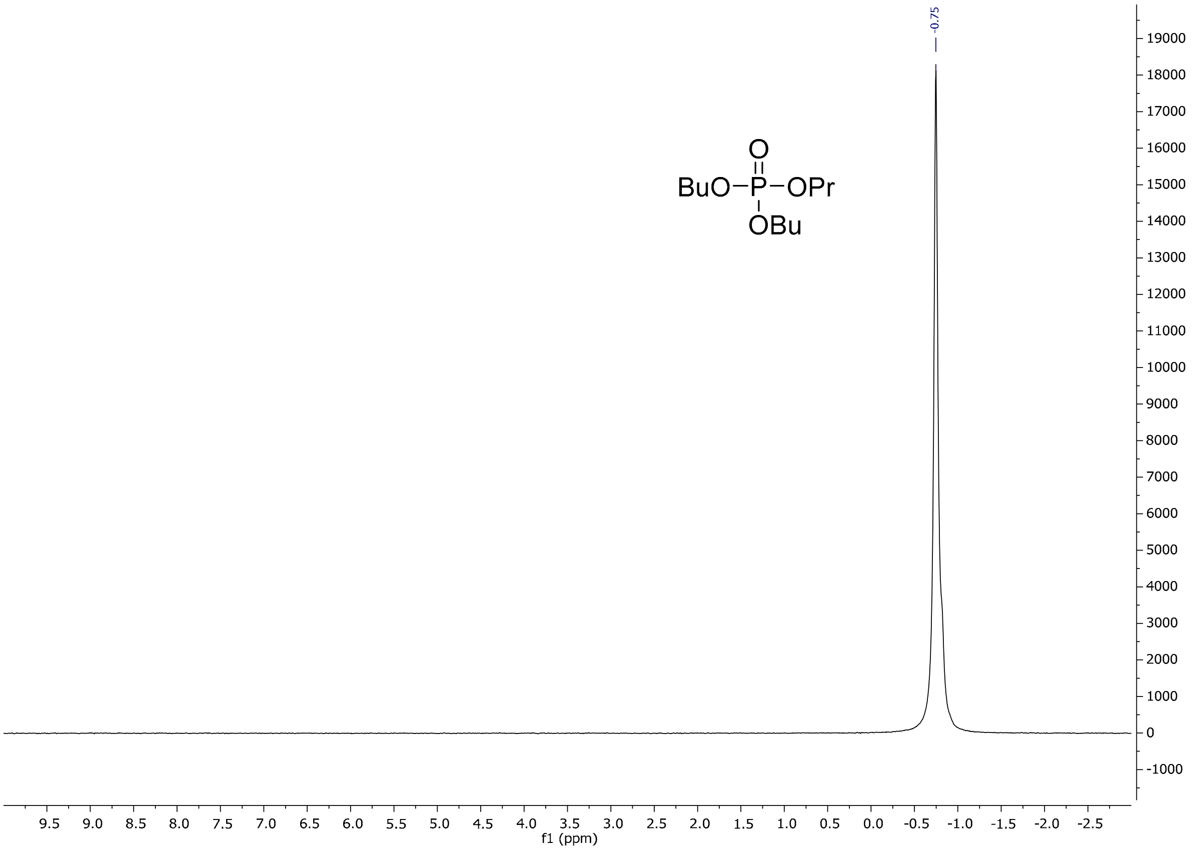

$^{13}C$ NMR (125.7 MHz, $CDCl_3$)

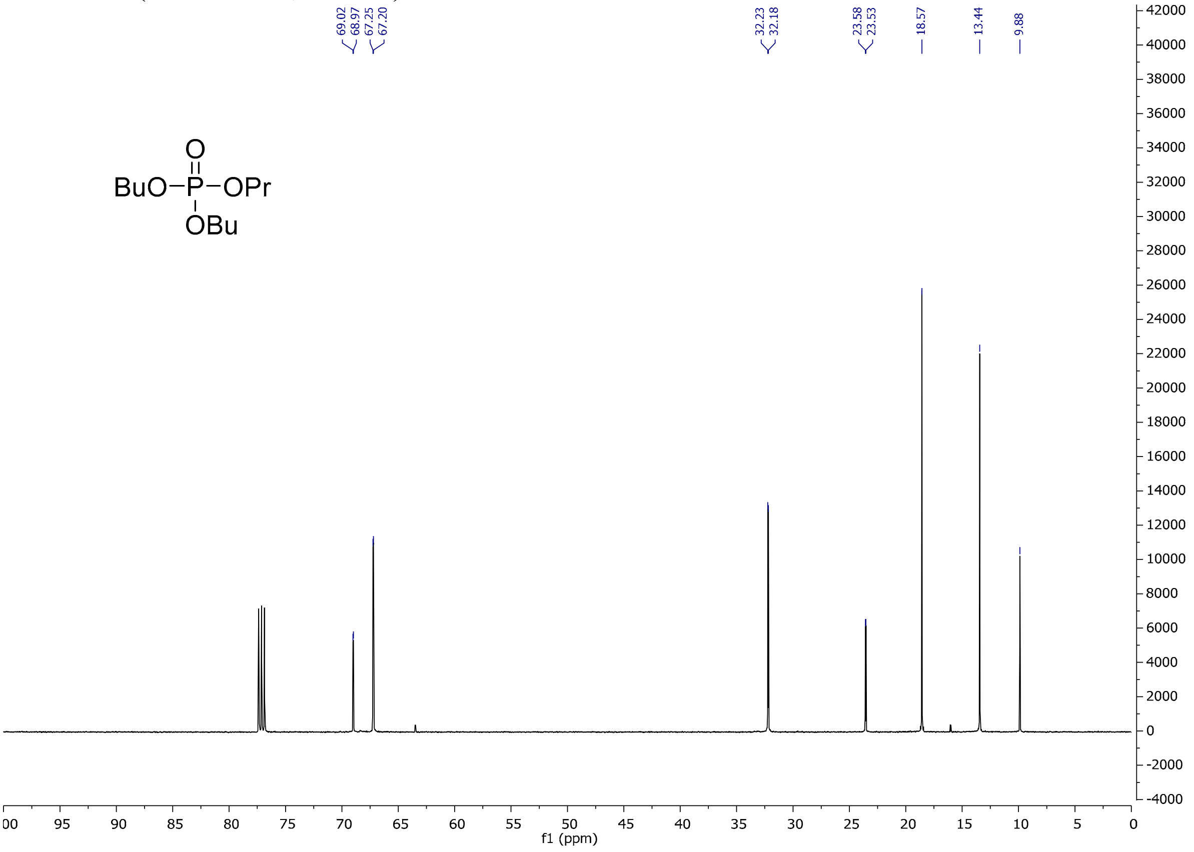


$^{1}H$ NMR (500 MHz, $CDCl_3$)

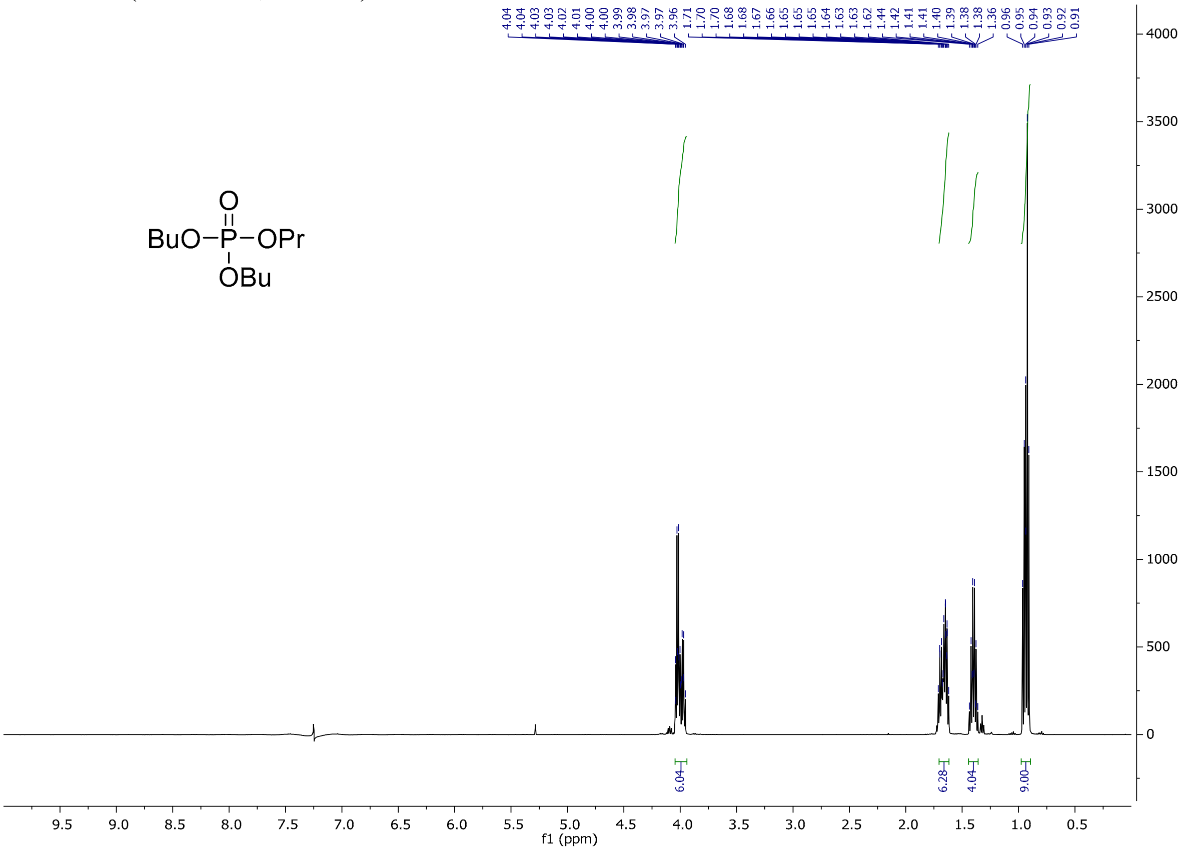

***Dibutyl-isopropylphosphate (7c)***

$^{31}P$ NMR (202.4 MHz, $CDCl_3$)

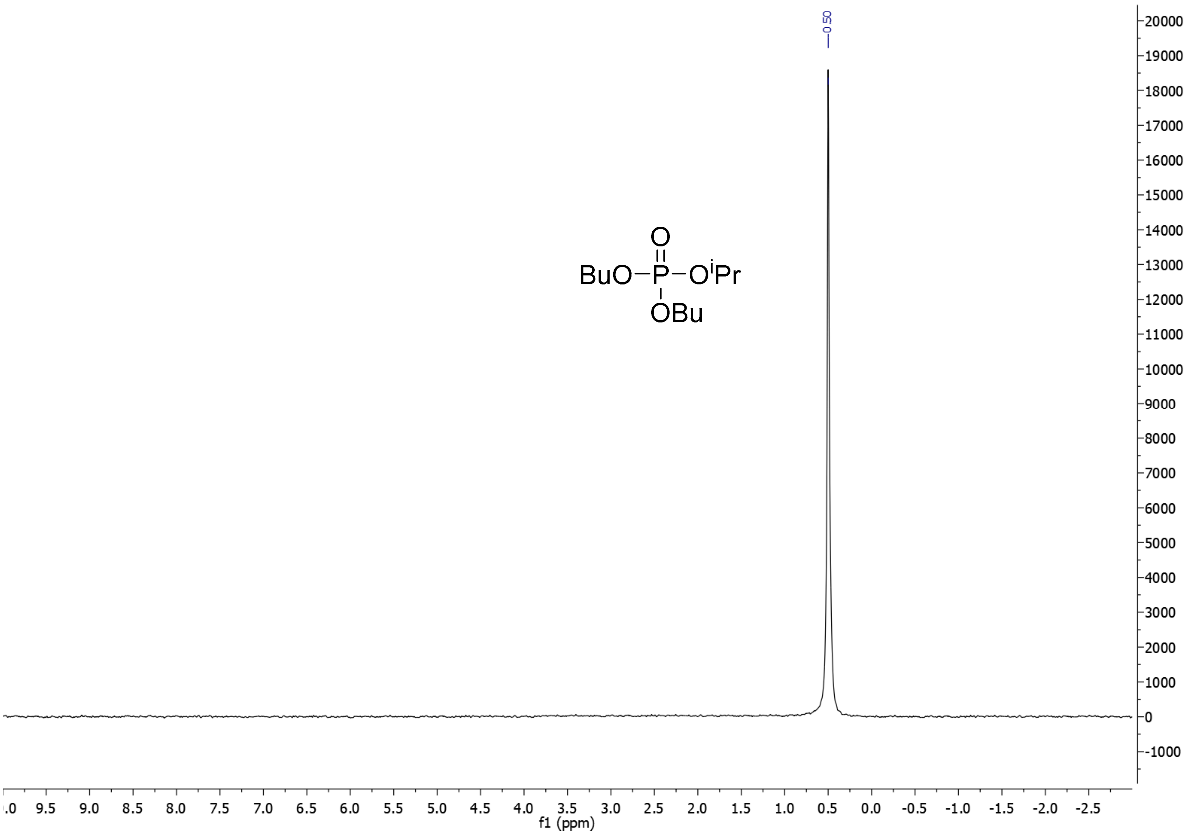

$^{13}$C NMR (125.7 MHz, $CDCl_3$)

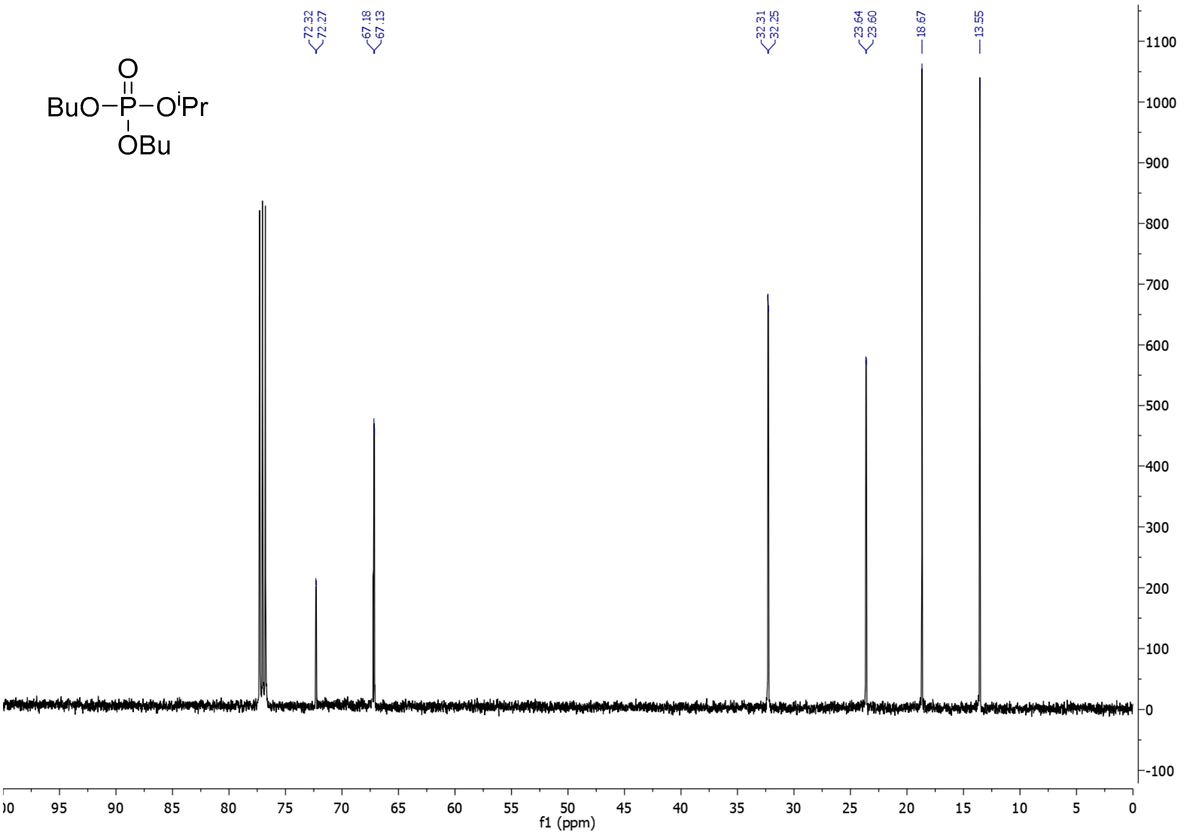


$^{1}$H NMR (500 MHz, $CDCl_3$)

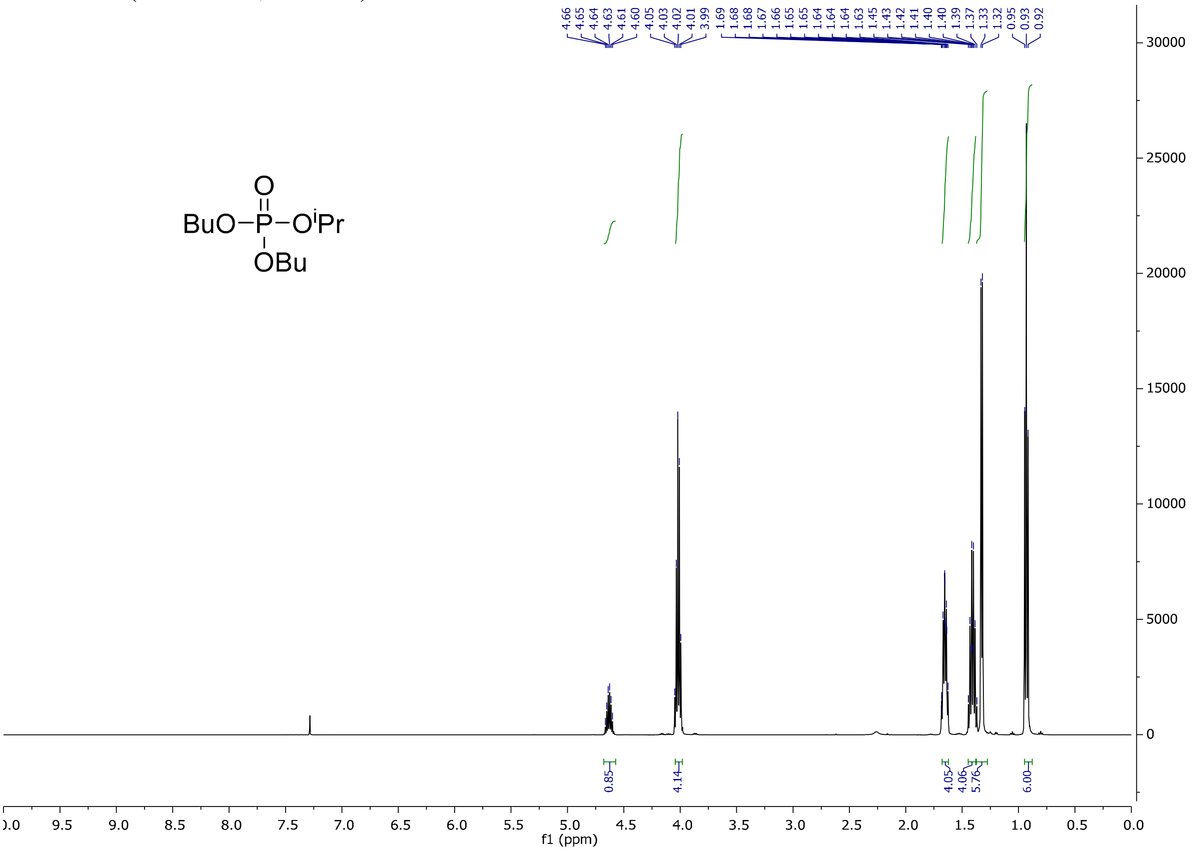

***Dibutyl-pentylphosphate (7d)***

$^{31}P$ NMR (202.4 MHz, $CDCl_3$)

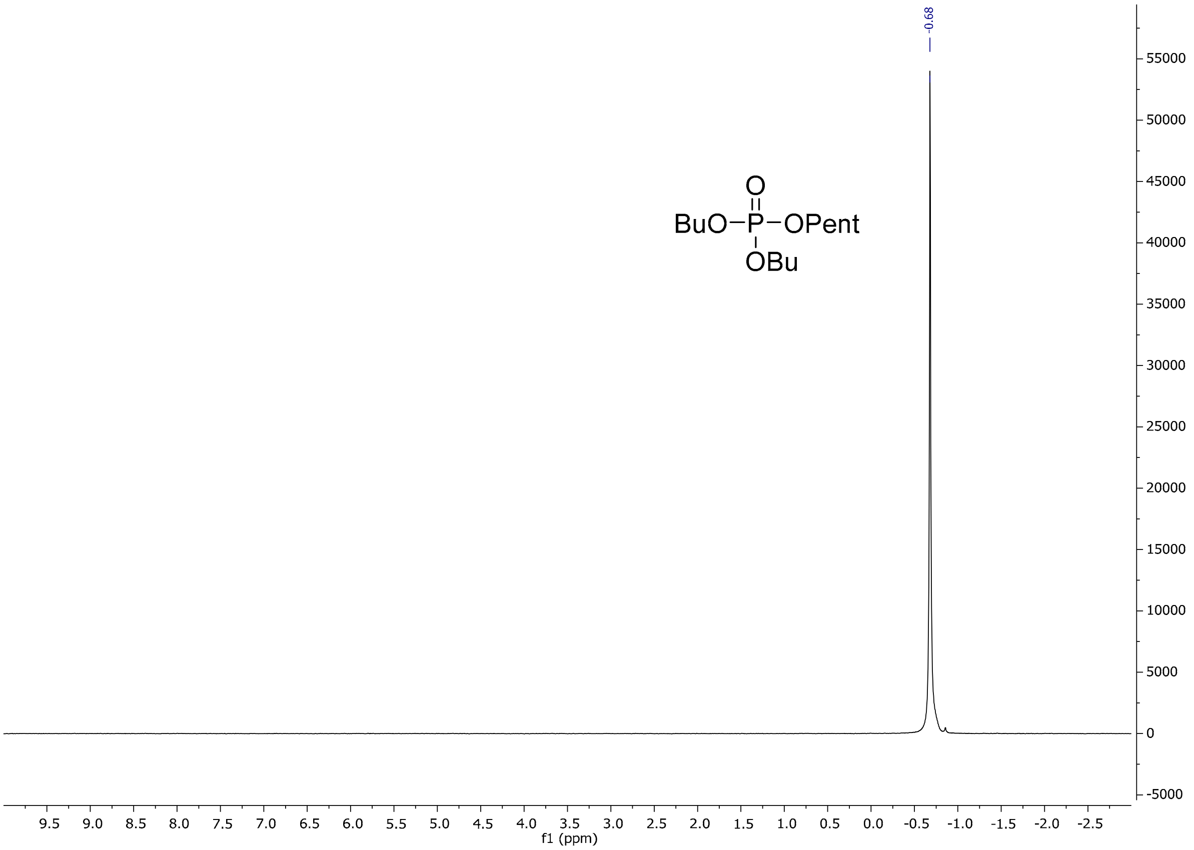

$^{13}C$ NMR (125.7 MHz, $CDCl_3$)

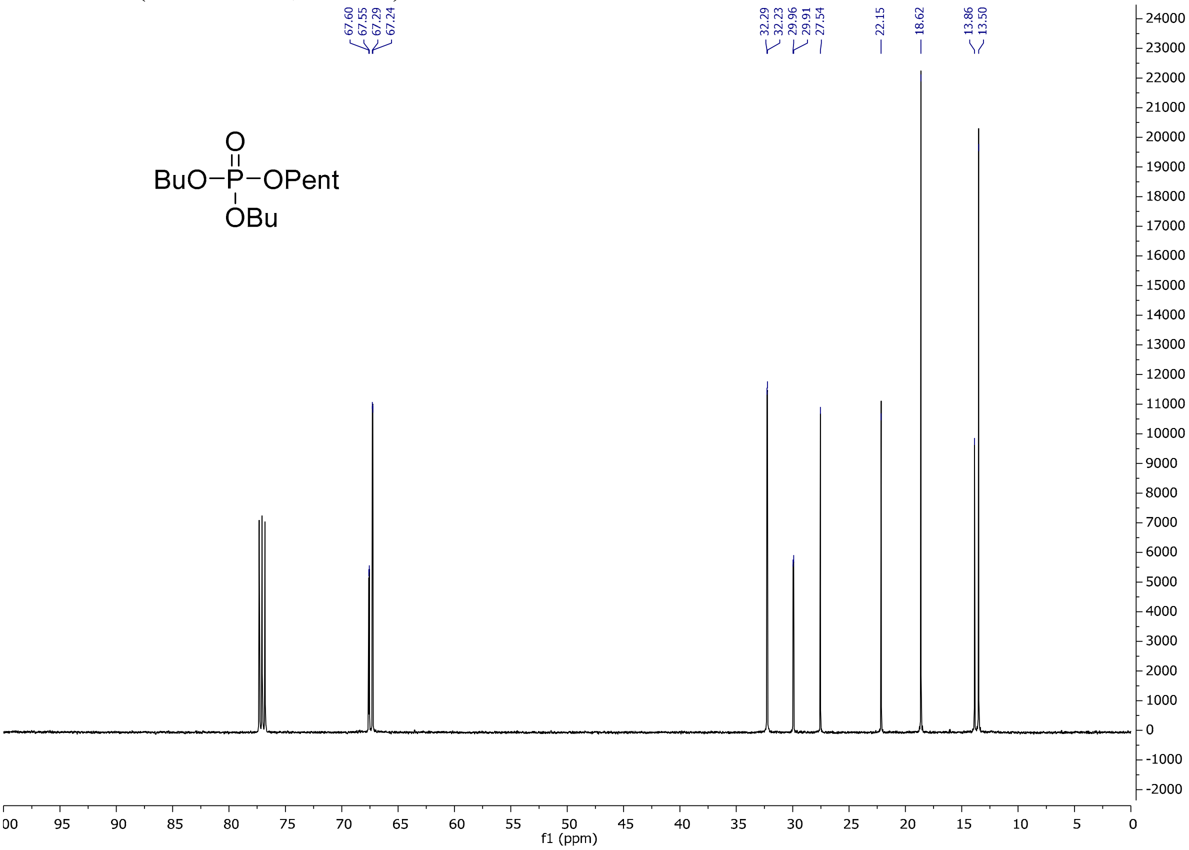


$^{1}H$ NMR (500 MHz, $CDCl_3$)

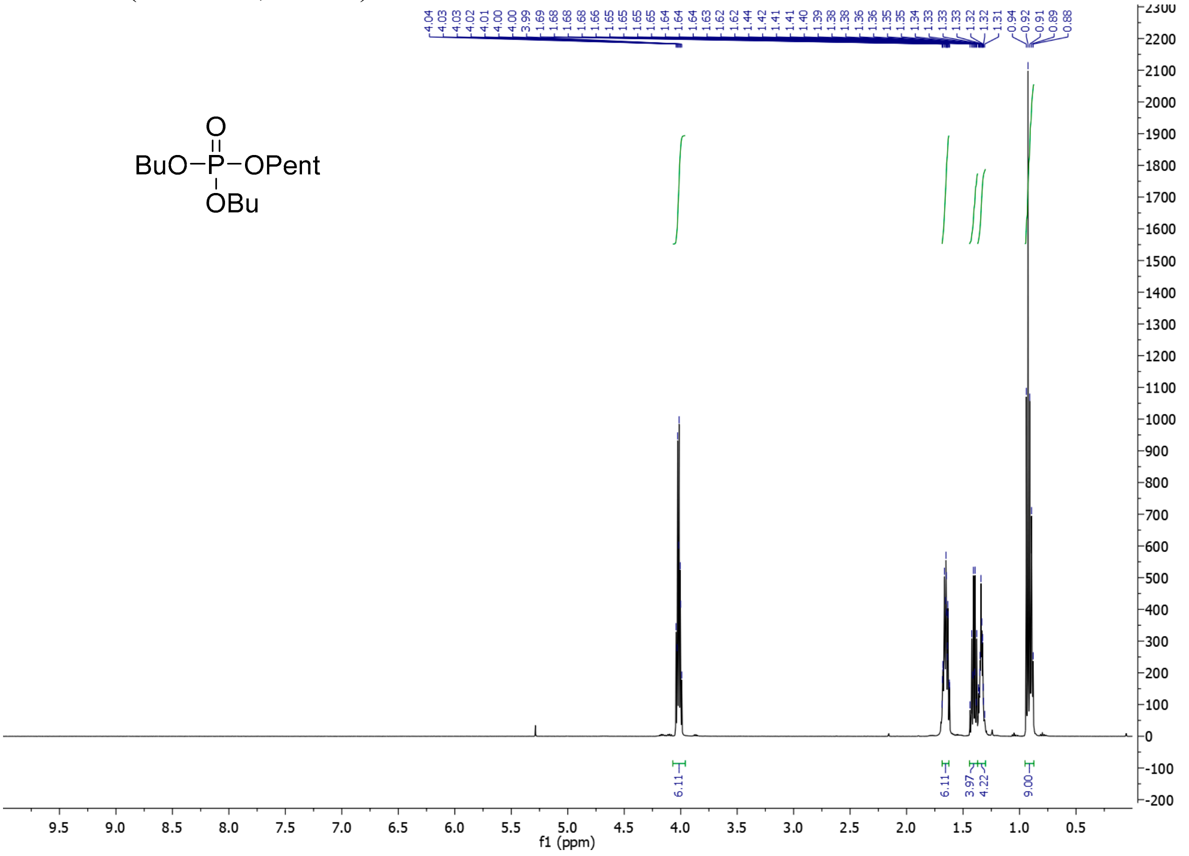

***Dipentyl-ethylphosphate (8a)***

$^{31}$P NMR (202.4 MHz, $CDCl_3$)

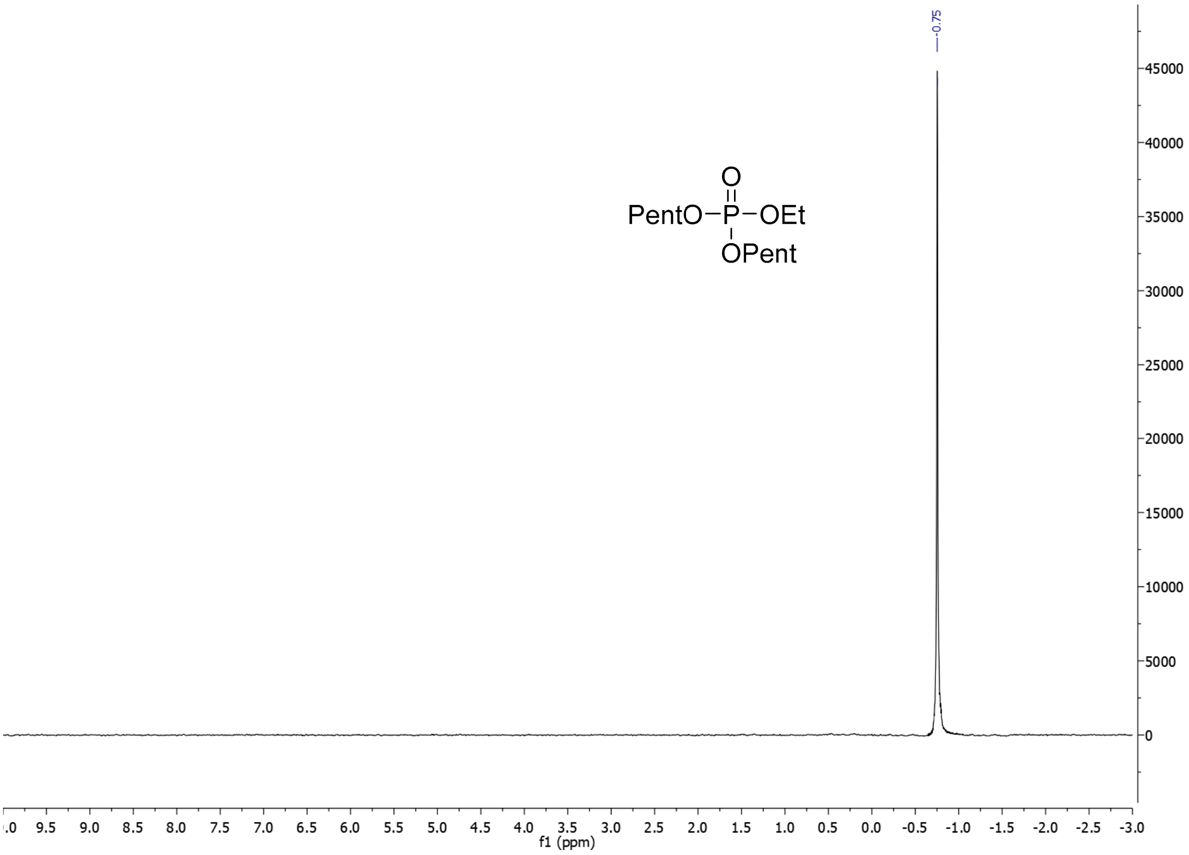

$^{13}C$ NMR (125.7 MHz, $CDCl_3$)

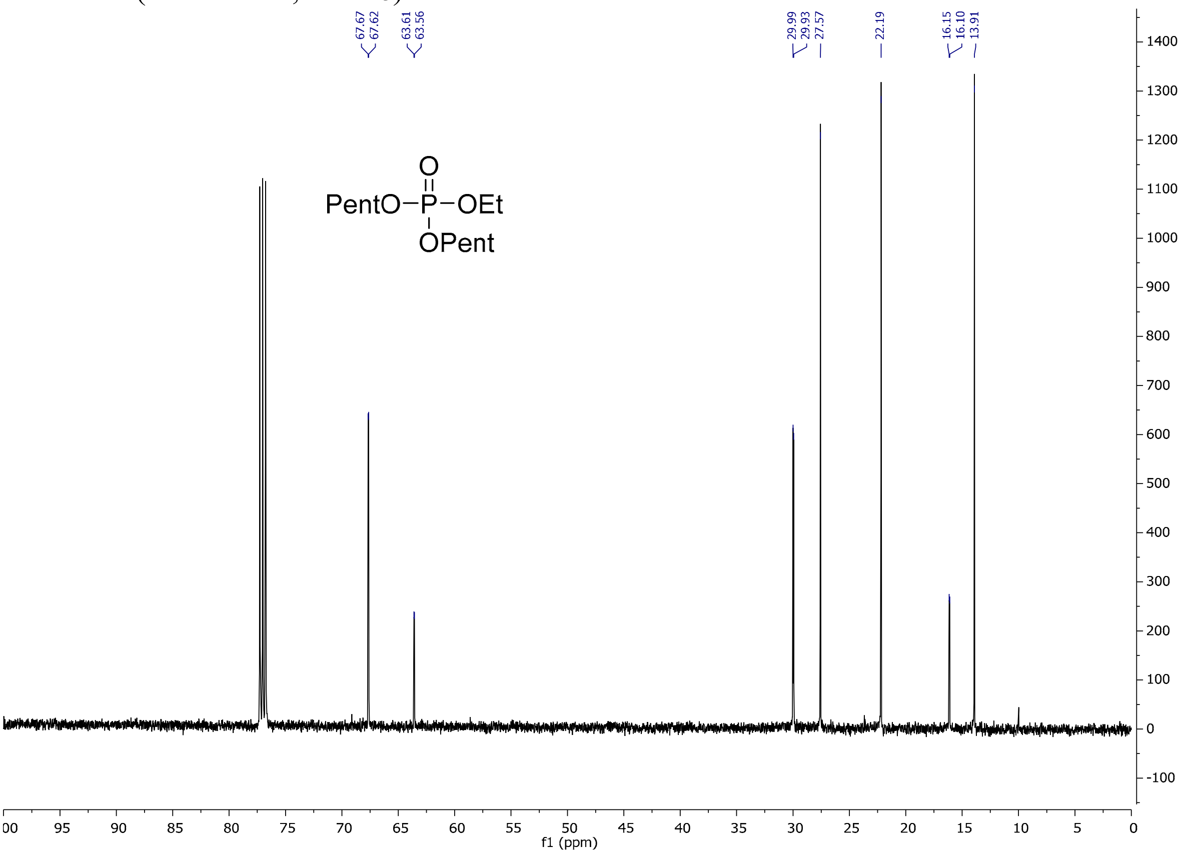


$^{1}H$ NMR (500 MHz, $CDCl_3$)

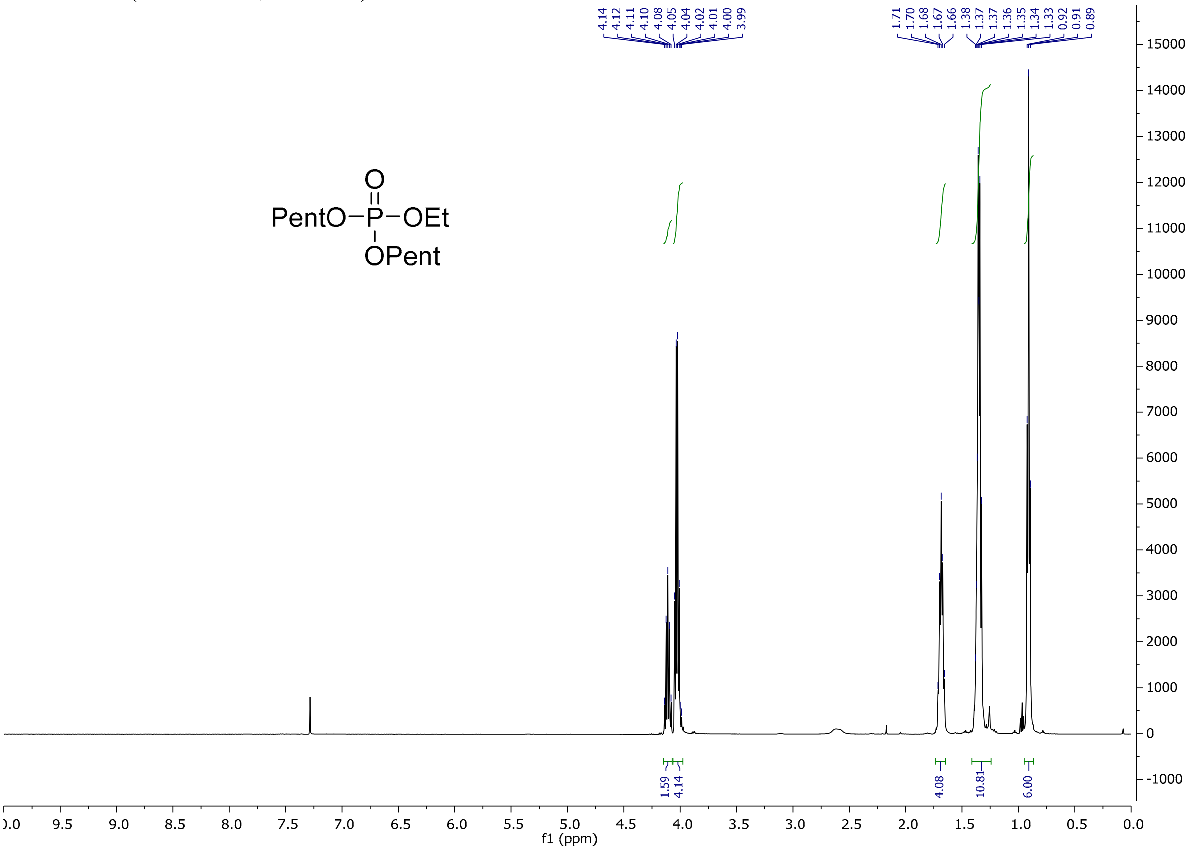

***Dipentyl-propylphosphate (8b)***

$^{31}P$ NMR (202.4 MHz, $CDCl_3$)

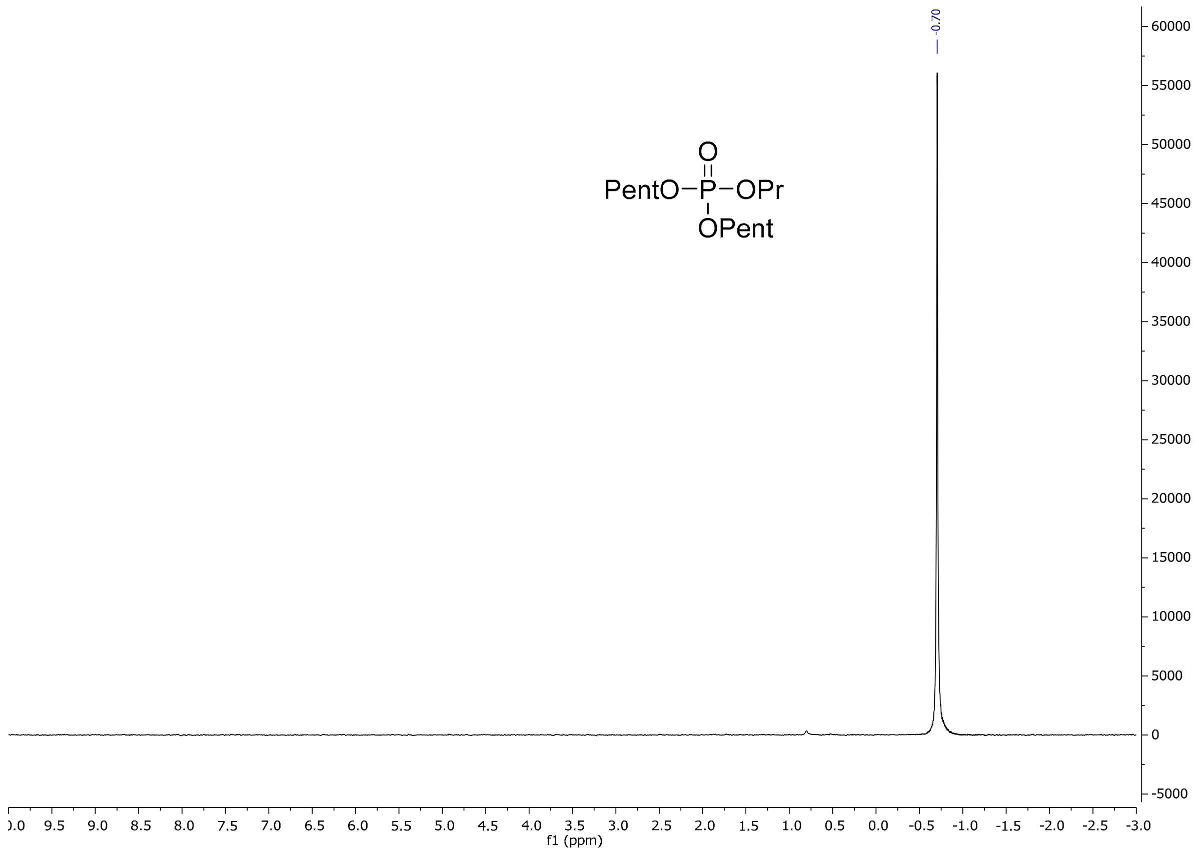

$^{13}C$ NMR (125.7 MHz, $CDCl_3$)

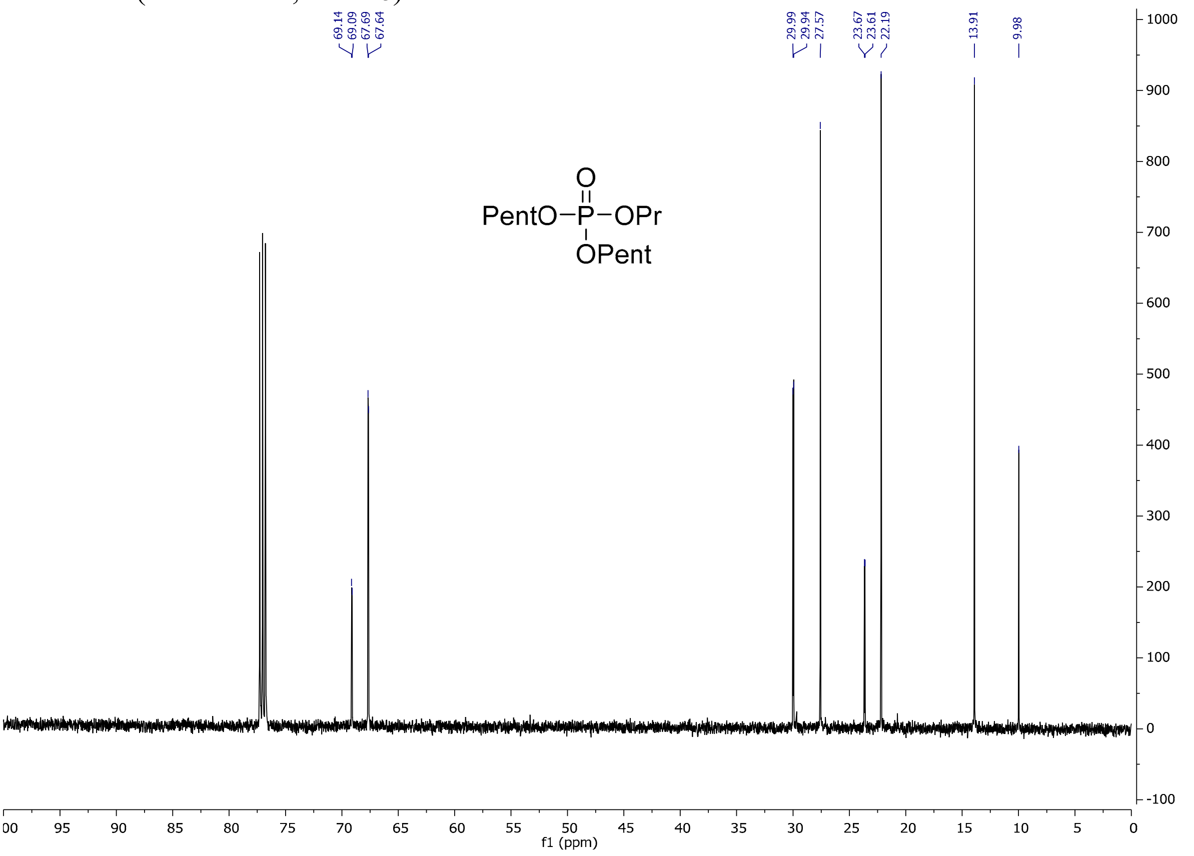


$^{1}H$ NMR (500 MHz, $CDCl_3$)

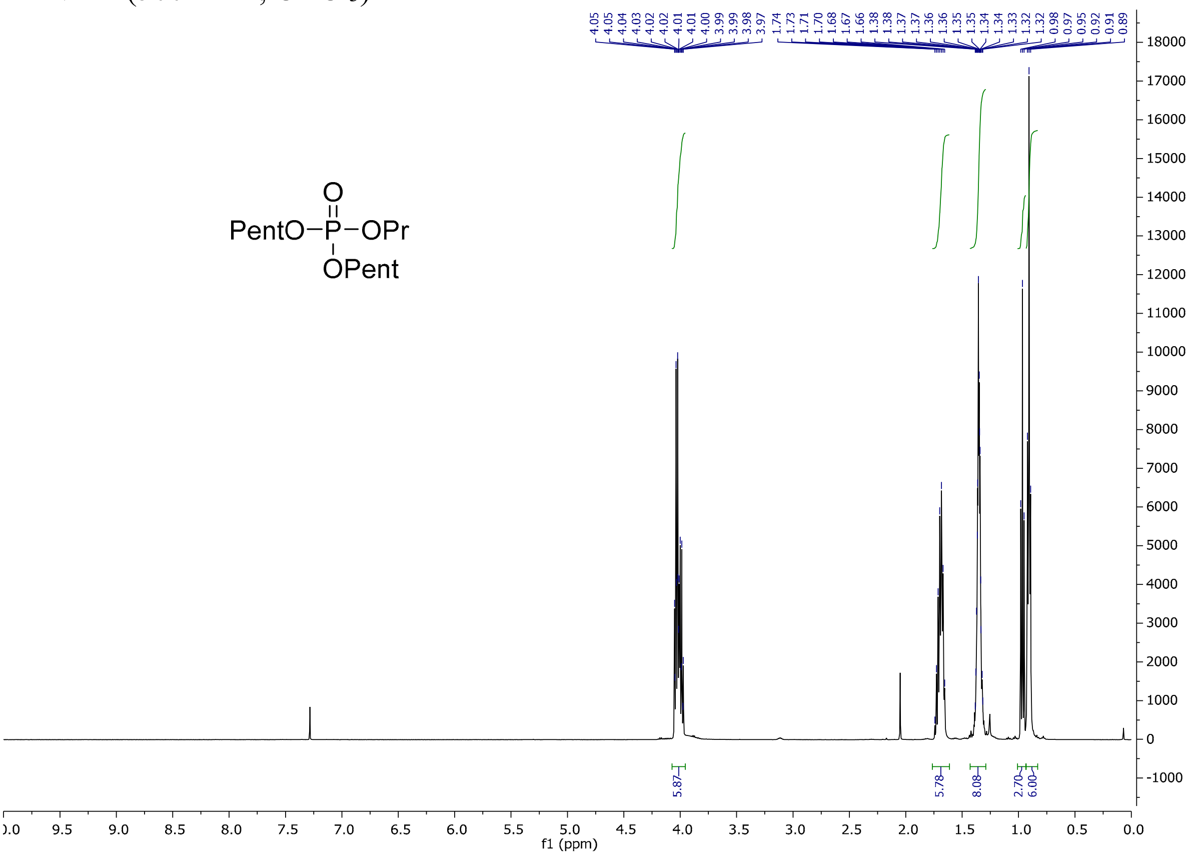

***Dipentyl-isopropylphosphate (8c)***

$^{31}$P NMR (202.4 MHz, $CDCl_3$)

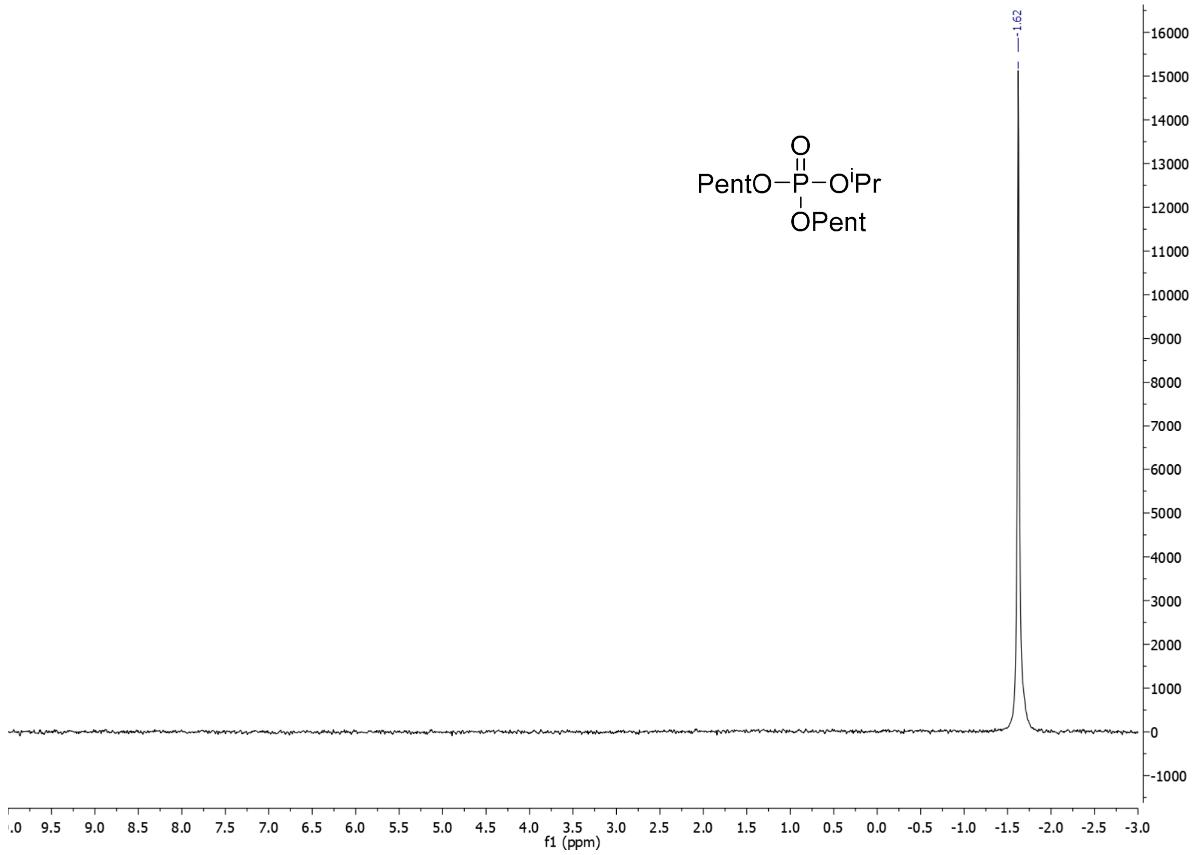

$^{13}C$ NMR (125.7 MHz, $CDCl_3$)

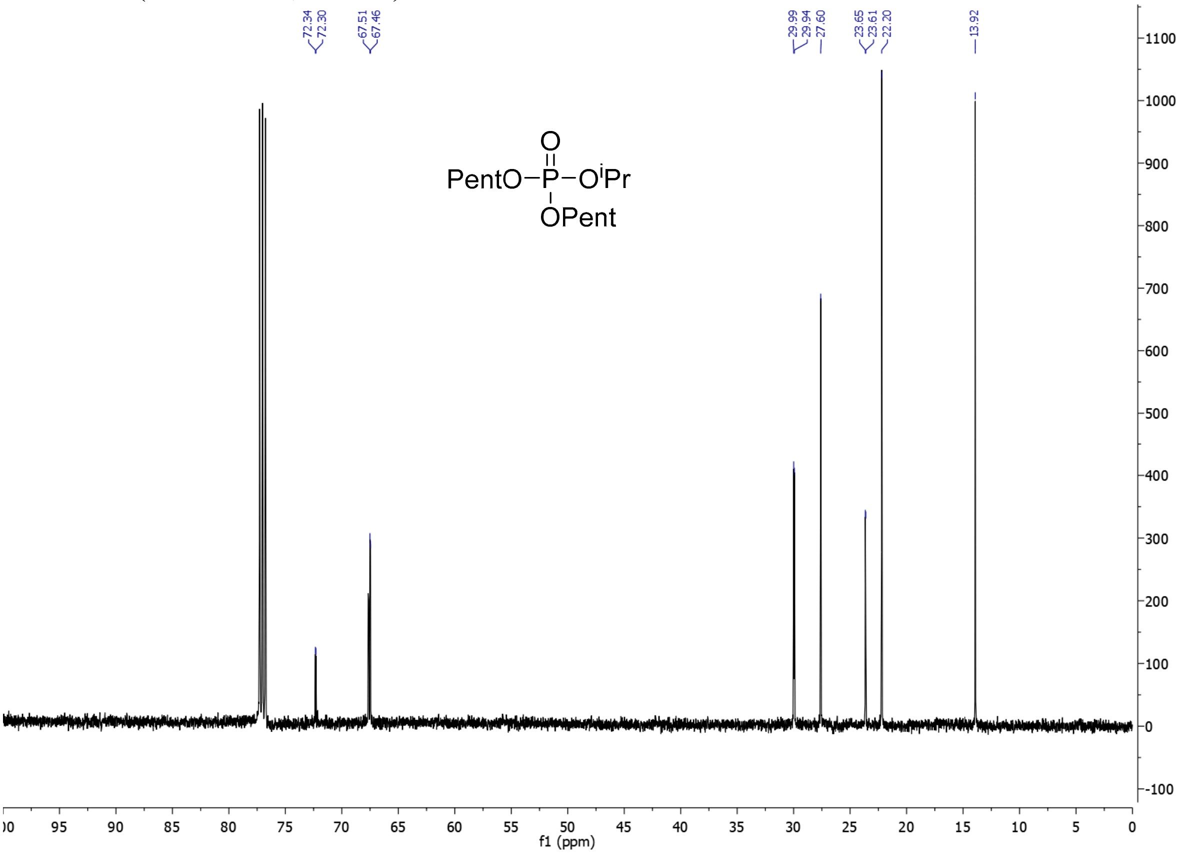


$^{1}H$ NMR (500 MHz, $CDCl_3$)

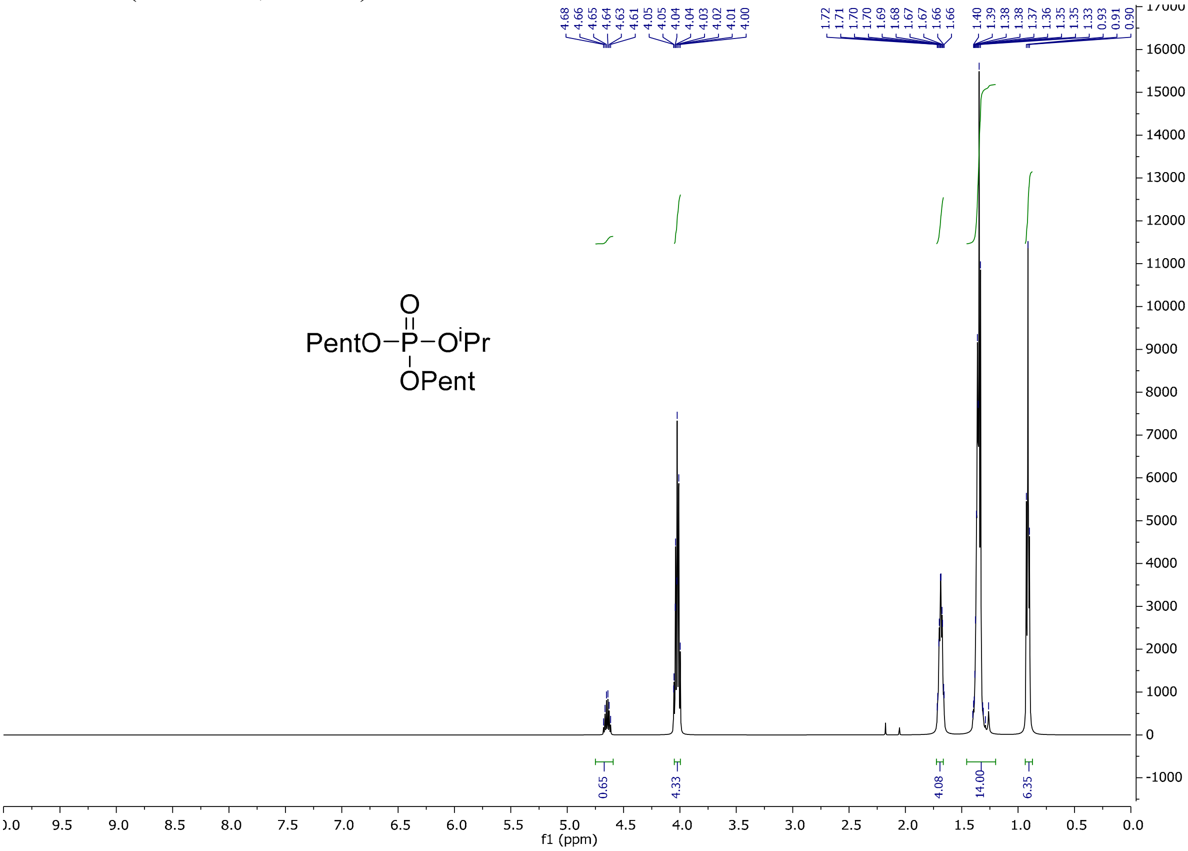

***Dipentyl-butylphosphate (8d)***

$^{31}P$ NMR (202.4 MHz, $CDCl_3$)

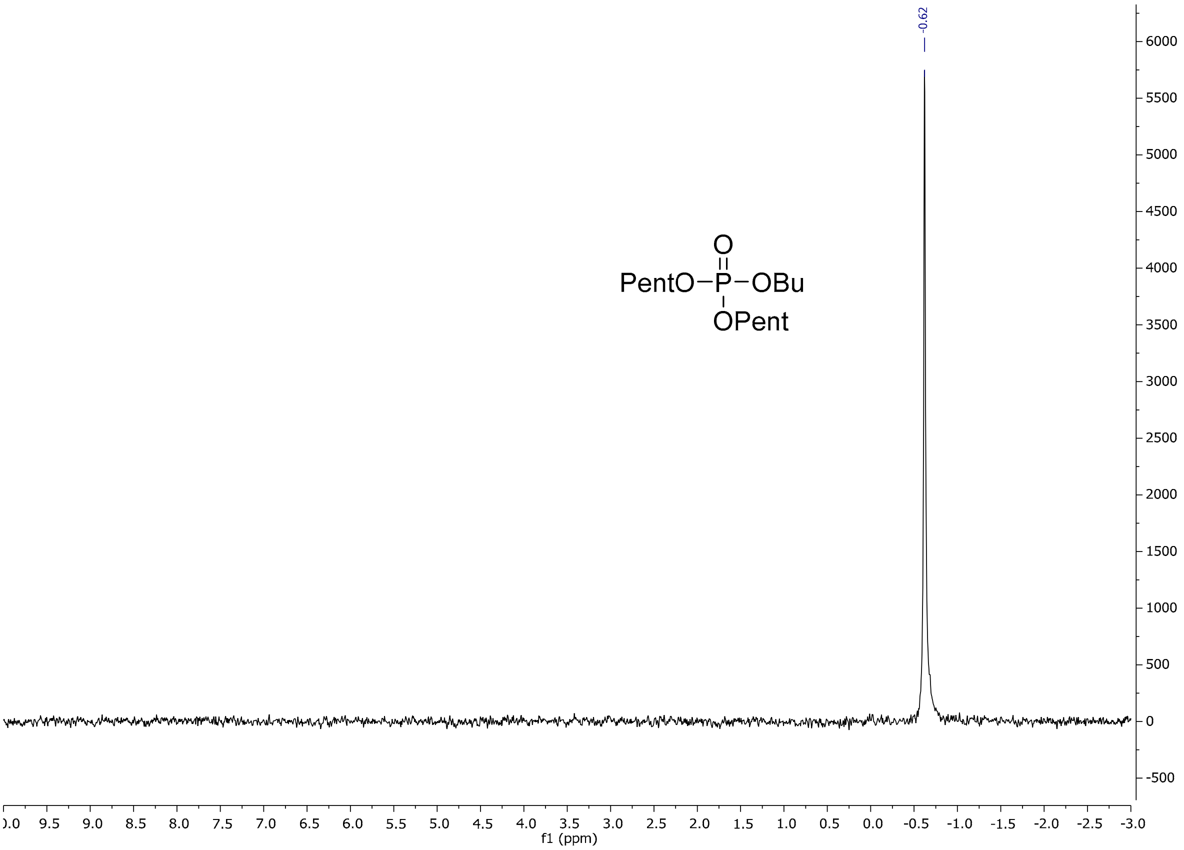

$^{13}C$ NMR (125.7 MHz, $CDCl_3$)

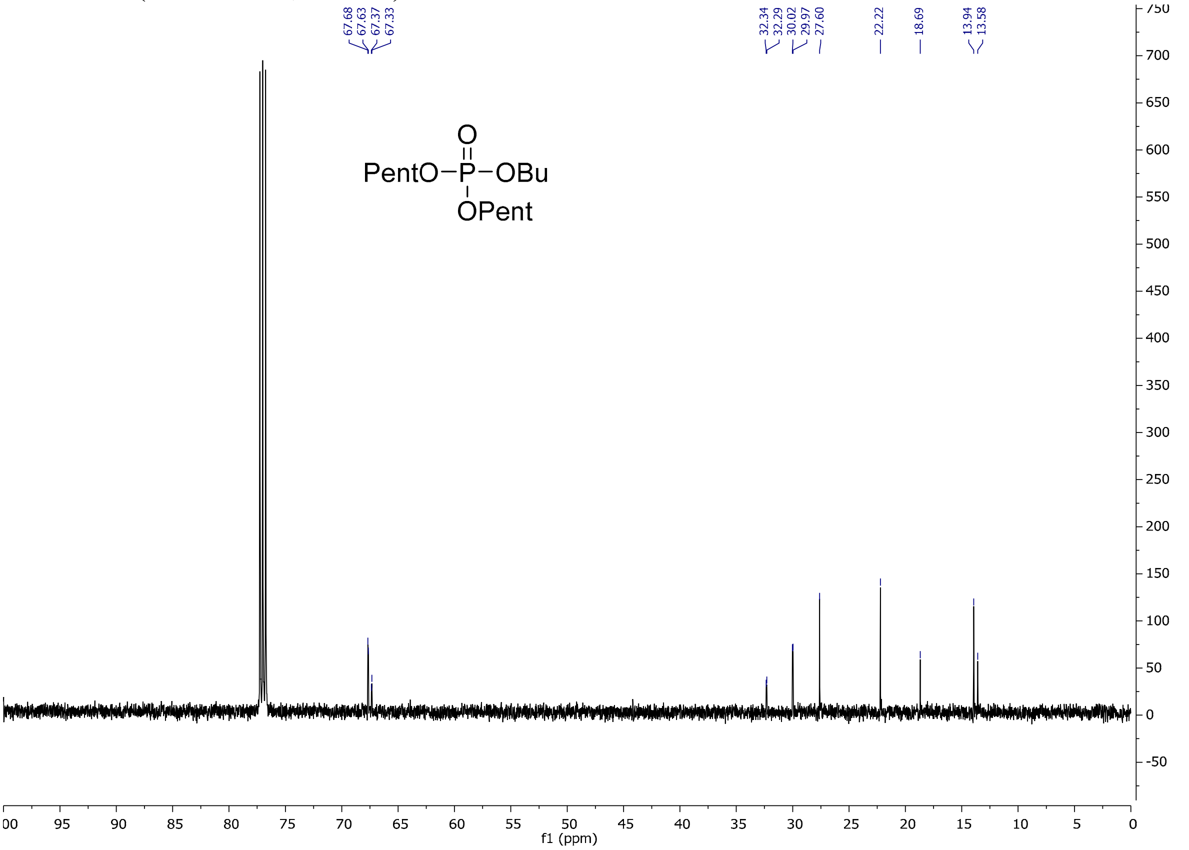


$^{1}H$ NMR (500 MHz, $CDCl_3$)

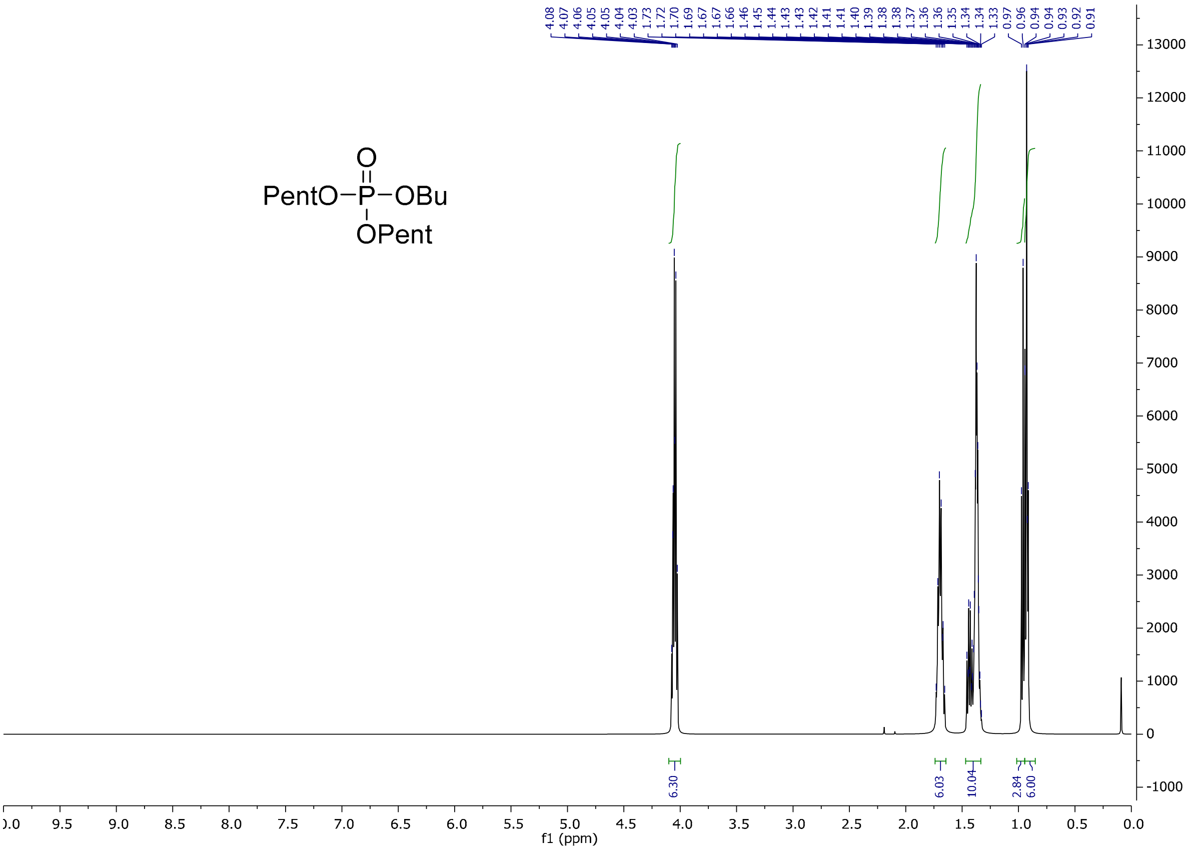

## Computed raw data

**Table S1.** Computed energies (E), zero point energies, internal energies (U), enthalpies (H) and Gibbs free energies (G) given in Hartree as well as entropies (S) given in Cal mol$^{-1}$ K$^{-1}$ at M06-2X/6-311+G(d,p) basis set considering an SMD solvent method using the parameter set of the appropriate alcohols for small molecules.

| $R^1$ | $R^2$ | Nr. | E | ZPE | U | H | G | S | $S_{vib}$ | Imaginary frequency |
|---|---|---|---|---|---|---|---|---|---|---|
| Et | OH | **4** | -1832.03936 | -1831.63504 | -1831.56924 | -1831.56774 | -1831.76164 | 257.2 | 175.1 | |
| | | **TS1** | -1832.00095 | -1831.59698 | -1831.53313 | -1831.53163 | -1831.71938 | 249.1 | 166.9 | -404.8 |
| | | **5** | -1832.00210 | -1831.59619 | -1831.53164 | -1831.53014 | -1831.72058 | 252.6 | 170.6 | |
| | | **TS2** | -1831.97390 | -1831.56989 | -1831.50566 | -1831.50416 | -1831.69239 | 249.7 | 167.7 | -274.26 |
| | | **6** | -1832.03718 | -1831.63607 | -1831.56876 | -1831.56726 | -1831.77024 | 269.3 | 186.8 | |
| | Et | **4** | -1989.21851 | -1988.70167 | -1988.62204 | -1988.62054 | -1988.85118 | 306.0 | 222.5 | |
| | | **TS1** | -1989.16998 | -1988.65756 | -1988.58459 | -1988.58310 | -1988.79323 | 278.8 | 195.3 | -435.19 |
| | | **5** | -1989.18797 | -1988.66776 | -1988.59145 | -1988.58995 | -1988.80168 | 280.9 | 197.7 | |
| | | **TS2** | -1989.14976 | -1988.63259 | -1988.55580 | -1988.55431 | -1988.77044 | 286.7 | 203.5 | -1457 |
| | | **6** | -1989.21540 | -1988.69712 | -1988.61659 | -1988.61509 | -1988.84543 | 305.6 | 222.3 | |
| Bu | OH | **4** | -2067.86444 | -2067.28842 | -2067.20432 | -2067.20282 | -2067.44100 | 316.0 | 231.4 | |
| | | **TS1** | -2067.82478 | -2067.24984 | -2067.16884 | -2067.16735 | -2067.39283 | 299.1 | 214.6 | -583.24 |
| | | **5** | -2067.82883 | -2067.25088 | -2067.16737 | -2067.16587 | -2067.40208 | 313.4 | 228.8 | |
| | | **TS2** | -2067.79419 | -2067.22143 | -2067.14246 | -2067.14096 | -2067.36295 | 294.5 | 209.9 | -268.25 |
| | | **6** | -2067.86565 | -2067.29051 | -2067.20601 | -2067.20451 | -2067.44532 | 319.5 | 234.8 | |
| | Bu | **4** | -2382.26215 | -2381.46013 | -2381.34976 | -2381.34826 | -2381.65304 | 404.3 | 317.7 | |
| | | **TS1** | -2382.21612 | -2381.41506 | -2381.30855 | -2381.30706 | -2381.59759 | 385.4 | 298.8 | -107.86 |
| | | **5** | -2382.22466 | -2381.41944 | -2381.31188 | -2381.31038 | -2381.60504 | 390.9 | 304.3 | |
| | | **TS2** | -2382.1973 | -2381.39386 | -2381.28544 | -2381.28394 | -2381.57748 | 389.4 | 302.9 | -245.46 |
| | | **6** | -2382.25350 | -2381.44981 | -2381.33899 | -2381.33749 | -2381.63956 | 400.7 | 314.3 | |

## Calculated data

Table S2. Calculated ΔG and ΔS values in kJ mol$^{-1}$ and J mol$^{-1}$ K$^{-1}$, respectively.

| $R^1$ | $R^2$ | Nr. | 2*(1 / 2) + 2 MeOH | 4 | TS1 | 5 | TS2 | 6 | 2 / 3 + 1 / 2 + $H_2O$ + 2 MeOH |
|---|---|---|---|---|---|---|---|---|---|
| Et | OH | **ΔG** | 0.0 | 165.6 | 276.5 | 265.4 | 339.3 | 143.0 | –4.9 |
| | | **ΔS** | 0.0 | 348.3 | 313.8 | 329.2 | 317.0 | 397.1 | 34.3 |
| | Et | **ΔG** | 0.0 | 158.1 | 310.1 | 280.0 | 369.9 | 181.1 | 7.4 |
| | | **ΔS** | 0.0 | 395.0 | 281.3 | 291.2 | 315.7 | 394.3 | 17.2 |
| Bu | OH | **ΔG** | 0.0 | 159.1 | 285.4 | 253.3 | 355.9 | 147.8 | –7.8 |
| | | **ΔS** | 0.0 | 375.2 | 305.0 | 364.2 | 147.8 | 389.5 | 42.5 |
| | Bu | **ΔG** | 0.0 | 156.3 | 301.8 | 274.3 | 346.6 | 191.7 | –15.4 |
| | | **ΔS** | 0.0 | 439.7 | 360.9 | 383.7 | 377.7 | 425.7 | 71.4 |

# XYZ geometries of computed species

**R = Et, OH, monoalkylation**

**4**

| | | | |
|---|---|---|---|
| P | 2.00738600 | -0.29008500 | 0.49584900 |
| P | -1.56068400 | 1.25114800 | -0.88786000 |
| O | 1.13200200 | 3.09905000 | 0.35254200 |
| H | 0.22853100 | 3.07490100 | -0.03495000 |
| O | -3.38466100 | -1.49505800 | -0.72833800 |
| H | -2.46895500 | -1.85718000 | -0.77908500 |
| O | -0.91831500 | -2.50019800 | -0.52148600 |
| H | -0.31148400 | -2.04938300 | 0.09510100 |
| O | 1.22333600 | -1.49965400 | 0.85006200 |
| O | -1.22260000 | 2.70351700 | -0.91293300 |
| O | 3.57978400 | -0.52576400 | 0.51987400 |
| O | 1.81878900 | 0.96620400 | 1.38603500 |
| H | 1.49640200 | 1.86545800 | 0.93691200 |
| O | 1.67063800 | 0.13109800 | -1.02810400 |
| H | 2.32664000 | 0.70714800 | -1.45180600 |
| O | -3.09308500 | 0.98888800 | -1.06417400 |
| H | -3.31129000 | -0.01211200 | -0.94385500 |
| O | -0.87601800 | 0.35922200 | -2.00565200 |
| O | -1.07520200 | 0.52749000 | 0.44721500 |
| C | -3.97603600 | -1.95892400 | 0.49855500 |
| C | -0.14242000 | -2.95586600 | -1.62506300 |
| C | 4.11669100 | -1.75899400 | -0.03500100 |
| C | -1.39063100 | 1.12358300 | 1.73540100 |
| C | 2.05281300 | 3.51824600 | -0.66239900 |
| H | 3.80236700 | -2.58360100 | 0.60704300 |
| H | 3.69741000 | -1.90430000 | -1.03453000 |
| H | 2.00569400 | 4.60178000 | -0.78206300 |
| H | 3.05399900 | 3.23276000 | -0.33819100 |
| H | 1.82235400 | 3.03463800 | -1.61493300 |
| H | 0.55286200 | -3.74056500 | -1.31276300 |
| H | -0.82973000 | -3.36558700 | -2.36612500 |
| H | 0.42347500 | -2.13779600 | -2.08042400 |
| H | -4.63311400 | -2.80082300 | 0.26427700 |
| H | -3.18091200 | -2.31981700 | 1.15670100 |
| C | -4.75318000 | -0.83876500 | 1.15402400 |
| H | -5.24202700 | -1.20799800 | 2.05853500 |
| H | -4.09264400 | -0.01326800 | 1.43062900 |
| H | -5.52370300 | -0.45782300 | 0.47836700 |
| H | -0.68171600 | 1.93409500 | 1.91507500 |
| H | -2.40067100 | 1.54073900 | 1.69576000 |
| C | 5.61736600 | -1.62343300 | -0.07992500 |
| H | 6.05002600 | -2.53946500 | -0.48794200 |
| H | 5.91060100 | -0.78633300 | -0.71660200 |
| H | 6.01928600 | -1.46636600 | 0.92299000 |
| C | -1.28247000 | 0.04135100 | 2.77921600 |
| H | -1.97552100 | -0.77451300 | 2.55891700 |
| H | -0.26657400 | -0.35527900 | 2.81761200 |
| H | -1.53301400 | 0.45718600 | 3.75794600 |
| H | 0.07104800 | 0.18601600 | -1.82160400 |

**TS1**

| | | | |
|---|---|---|---|
| P | 2.22274800 | -0.06396000 | 0.29770500 |
| P | -1.99806900 | 0.27289800 | -0.68343200 |
| O | 0.30601400 | 2.98007700 | -0.62905600 |
| H | -1.10936600 | 2.29349900 | -1.02412100 |
| O | -2.11428300 | -1.49629800 | -0.10679500 |
| H | -1.12957800 | -2.12064200 | -0.18508600 |
| O | -0.13885200 | -2.83632900 | -0.21396900 |
| H | 0.64009900 | -2.28092500 | 0.14436000 |
| O | 1.80886100 | -1.48494200 | 0.59490100 |
| O | -1.92192700 | 1.77963500 | -1.29346400 |
| O | 3.73111000 | 0.17124700 | 0.81315500 |
| O | 1.41091600 | 1.07932500 | 0.83866400 |
| H | 0.74142300 | 2.30152900 | -0.05761500 |
| O | 2.33771100 | 0.02165200 | -1.31984000 |
| H | 2.70025100 | 0.86082400 | -1.63774600 |
| O | -3.34595300 | 0.00485100 | -1.50873600 |
| H | -3.64602200 | -0.91181500 | -1.45231200 |
| O | -0.59465900 | -0.25584000 | -1.26288200 |
| O | -2.06211000 | 0.63015700 | 0.86749700 |
| C | -2.84076600 | -1.92636400 | 1.06912700 |
| C | 0.17231100 | -3.25149900 | -1.55120400 |
| C | 4.73703400 | -0.83378100 | 0.56169900 |
| C | -2.27861700 | 1.95331300 | 1.42100500 |
| C | 1.10166900 | 3.17077600 | -1.79568700 |
| H | 4.48155300 | -1.73034500 | 1.13108600 |
| H | 4.73551400 | -1.08160700 | -0.50394400 |
| H | 0.76454200 | 4.08400800 | -2.28719400 |
| H | 2.15647500 | 3.28373300 | -1.52972800 |
| H | 0.99069500 | 2.33614700 | -2.49736600 |
| H | 0.78313400 | -4.15386700 | -1.51305000 |
| H | -0.76478100 | -3.46628600 | -2.06504100 |
| H | 0.70792500 | -2.46025500 | -2.07822600 |
| H | -2.69211400 | -3.00762100 | 1.10775700 |
| H | -2.38004600 | -1.47704700 | 1.94941000 |
| C | -4.31633900 | -1.60034700 | 0.99211500 |
| H | -4.80460800 | -1.99734200 | 1.88524900 |
| H | -4.49621200 | -0.52351600 | 0.96192900 |
| H | -4.78134400 | -2.06913800 | 0.12007900 |
| H | -1.41889600 | 2.58061500 | 1.18181400 |
| H | -3.17617900 | 2.38084300 | 0.97160400 |
| C | 6.07100900 | -0.27103400 | 0.98901800 |
| H | 6.85235100 | -1.01703500 | 0.82710600 |
| H | 6.31764500 | 0.62164200 | 0.41015600 |
| H | 6.05509000 | -0.01131200 | 2.04976900 |
| C | -2.43056300 | 1.77186200 | 2.91099900 |
| H | -3.28775300 | 1.13423900 | 3.13865900 |
| H | -1.52985100 | 1.32406400 | 3.33610600 |
| H | -2.58783000 | 2.74657000 | 3.37840300 |
| H | -0.12135500 | 0.40116000 | -1.79438800 |

**5**

| | | | |
|---|---|---|---|
| P | 2.11378400 | 0.09693700 | 0.25645600 |
| P | -2.16759900 | 0.23398300 | -0.55152000 |
| O | 0.04725300 | 3.09601300 | -0.61287500 |
| H | -1.45257500 | 2.35585200 | -0.76665300 |
| O | -2.07701400 | -1.51242100 | -0.32613000 |
| H | -0.81006500 | -2.13315500 | -0.77066000 |
| O | 0.10901200 | -2.59200200 | -0.86710700 |
| H | 0.79582700 | -1.98269600 | -0.23843500 |
| O | 1.53448400 | -1.28089200 | 0.54815300 |
| O | -2.29502200 | 1.84865800 | -0.88092200 |
| O | 3.61500400 | 0.14014900 | 0.81755100 |
| O | 1.41620600 | 1.30995600 | 0.78918800 |
| H | 0.56378200 | 2.45979000 | -0.06794700 |
| O | 2.25297700 | 0.13861300 | -1.35188500 |
| H | 2.54491900 | 0.99258900 | -1.70206900 |

| | | | |
|---|---|---|---|
| O | -3.71275300 | 0.08566200 | -0.99902900 |
| H | -3.99157400 | -0.83808200 | -1.03939400 |
| O | -0.97828400 | -0.02643400 | -1.62274400 |
| O | -1.76128800 | 0.38279600 | 0.99141000 |
| C | -2.42394900 | -2.18665800 | 0.89965800 |
| C | 0.54214400 | -2.67555800 | -2.23779500 |
| C | 4.50937400 | -0.96949600 | 0.57307900 |
| C | -1.89465100 | 1.56993400 | 1.80429000 |
| C | 0.76351000 | 3.32668800 | -1.82089800 |
| H | 4.15946600 | -1.82830900 | 1.15015000 |
| H | 4.47978800 | -1.22183600 | -0.49089600 |
| H | 0.25384800 | 4.12111600 | -2.36691300 |
| H | 1.78785900 | 3.64696300 | -1.60895000 |
| H | 0.79139700 | 2.43063000 | -2.45046000 |
| H | 1.47649000 | -3.23414700 | -2.25021600 |
| H | -0.21938300 | -3.21395800 | -2.79974600 |
| H | 0.68744600 | -1.67868700 | -2.65098400 |
| H | -2.27999500 | -3.24721800 | 0.67982600 |
| H | -1.71397500 | -1.89695600 | 1.67697500 |
| C | -3.84971000 | -1.94413700 | 1.35433400 |
| H | -4.03185600 | -2.53080400 | 2.25822800 |
| H | -4.03096800 | -0.89372400 | 1.59334100 |
| H | -4.56835500 | -2.26791400 | 0.59607300 |
| H | -1.19910900 | 2.33210000 | 1.45032000 |
| H | -2.91460700 | 1.94860300 | 1.71486000 |
| C | 5.89454400 | -0.54552600 | 0.99686700 |
| H | 6.59560200 | -1.36678500 | 0.83301300 |
| H | 6.22702300 | 0.31739300 | 0.41608700 |
| H | 5.90767600 | -0.28497800 | 2.05740100 |
| C | -1.57400900 | 1.15677800 | 3.22060000 |
| H | -2.27673900 | 0.39780900 | 3.57238800 |
| H | -0.55952800 | 0.75599600 | 3.27952900 |
| H | -1.64284600 | 2.02624700 | 3.87834200 |
| H | -0.78044100 | 0.75743300 | -2.15293600 |

**TS2**

| | | | |
|---|---|---|---|
| P | -1.78018100 | -0.56294200 | -0.34507200 |
| P | 2.28677300 | 0.27652700 | 0.07295100 |
| O | -0.58338400 | 3.05172300 | -1.69643000 |
| H | 1.07884100 | 2.37002400 | -1.15806000 |
| O | 2.79417700 | -0.90691500 | 1.11242600 |
| H | 1.31867400 | -1.56009200 | 1.87474100 |
| O | 0.46889400 | -2.00458900 | 2.07087700 |
| H | -0.35563100 | -1.84216600 | 0.90930800 |
| O | -0.96512000 | -1.82453700 | 0.05767300 |
| O | 1.83782200 | 1.76243500 | -1.18492800 |
| O | -3.13331800 | -1.12286800 | -0.97512200 |
| O | -1.16773500 | 0.38483400 | -1.31494900 |
| H | -1.00485300 | 2.18135300 | -1.56876000 |
| O | -2.14695700 | 0.10747400 | 1.05762000 |
| O | 3.58546400 | 1.14270100 | -0.01125400 |
| O | 1.12763300 | 0.66858700 | 1.09172800 |
| O | 1.87462900 | -0.75802000 | -1.05814200 |
| C | 3.54057600 | -2.06194100 | 0.67640900 |
| C | -0.09349600 | -1.42862000 | 3.25490100 |
| C | -3.89932100 | -2.14463600 | -0.28665100 |
| C | 1.75273000 | -0.57796100 | -2.48851600 |
| C | -1.34560600 | 4.06582400 | -1.05554300 |
| H | -3.34623700 | -3.08377700 | -0.35141000 |
| H | -4.00031300 | -1.86242300 | 0.76535100 |
| H | -0.87153400 | 5.02257300 | -1.27733900 |
| H | -2.37210400 | 4.08807800 | -1.43301400 |
| H | -1.36744500 | 3.93335900 | 0.03174100 |
| H | -1.10389500 | -1.82134500 | 3.36469600 |
| H | 0.50114900 | -1.71643800 | 4.12390800 |
| H | -0.12710100 | -0.34009400 | 3.17582700 |
| H | 3.77202800 | -2.59541200 | 1.59940100 |
| H | 2.88591700 | -2.69049900 | 0.06821600 |
| C | 4.81336500 | -1.72315500 | -0.07108100 |
| H | 5.35422200 | -2.65204400 | -0.26987900 |
| H | 4.60790400 | -1.24101900 | -1.02986500 |
| H | 5.45582100 | -1.07055300 | 0.52387600 |
| H | 0.92713700 | 0.10005400 | -2.68974900 |
| H | 2.68040100 | -0.14009600 | -2.86191800 |
| C | -5.24467700 | -2.24639700 | -0.96103900 |
| H | -5.83156400 | -3.03039000 | -0.47730400 |
| H | -5.79151200 | -1.30446800 | -0.88402900 |
| H | -5.12707800 | -2.50226000 | -2.01597500 |
| C | 1.50462500 | -1.94884400 | -3.07011000 |
| H | 2.33209400 | -2.62640800 | -2.84751600 |
| H | 0.58021100 | -2.37213300 | -2.66981400 |
| H | 1.40595000 | -1.86593500 | -4.15515000 |
| C | 0.43738000 | 1.91758900 | 1.31140200 |
| H | -0.21666700 | 1.69233400 | 2.15354200 |
| H | -0.18601600 | 2.12743000 | 0.44519200 |
| C | 1.36504400 | 3.06072700 | 1.65992300 |
| H | 2.02180900 | 2.77887700 | 2.48643000 |
| H | 1.97582800 | 3.37664700 | 0.81303300 |
| H | 0.76109600 | 3.91345600 | 1.98044100 |
| C | -2.88935600 | 1.35463300 | 1.09827500 |
| H | -2.54462400 | 1.86028900 | 1.99997000 |
| H | -2.61570600 | 1.95927400 | 0.22929800 |
| C | -4.37998400 | 1.09578300 | 1.14265900 |
| H | -4.71986800 | 0.60188800 | 0.22985700 |
| H | -4.63683800 | 0.47842700 | 2.00589900 |
| H | -4.90455900 | 2.05018400 | 1.23327500 |
| H | 2.96105800 | 1.89489700 | -0.70218300 |

**6**

| | | | |
|---|---|---|---|
| P | 2.03520600 | -0.39241200 | -0.16891300 |
| P | -2.23232900 | 0.26303600 | -0.21684300 |
| O | 4.10505900 | 1.19694800 | 2.21161400 |
| H | 0.29431400 | 0.47057400 | 0.84888900 |
| O | -1.82081800 | -0.97035400 | -1.11431700 |
| H | -0.83731100 | 2.91312400 | -2.10696400 |
| O | 0.75657900 | 2.50872200 | -1.98474900 |
| H | 0.90649500 | 1.54300600 | -1.94334600 |
| O | 1.46159500 | -0.07887300 | -1.51037400 |
| O | -1.08929400 | 0.93846500 | 0.48241100 |
| O | 2.04778900 | -1.95858100 | 0.13503200 |
| O | 1.25308400 | 0.19656700 | 1.05835700 |
| H | 3.78985500 | 0.71684400 | 2.98735600 |
| O | 3.51782800 | 0.11606900 | -0.02158300 |
| H | 3.77538000 | 0.47683300 | 0.89173500 |
| O | -3.03485300 | 1.19889800 | -1.16235300 |
| H | -2.52010000 | 2.02527500 | -1.55633700 |
| O | -1.80958700 | 3.12164000 | -2.13014500 |
| O | -3.27647000 | -0.37120200 | 0.79756100 |
| C | -1.14826300 | -2.12985200 | -0.54332300 |
| C | 1.33272800 | 3.07322600 | -0.80726500 |
| C | 2.51618400 | -2.88736000 | -0.87406500 |
| C | -3.76213400 | 0.41801400 | 1.91786000 |

| | | | |
|---|---|---|---|
| C | 3.54977400 | 2.51884200 | 2.21443700 |
| H | 1.99510900 | -2.67527900 | -1.81086700 |
| H | 3.58826700 | -2.72912900 | -1.01865100 |
| H | 3.94275600 | 3.02728500 | 1.33489800 |
| H | 2.45936100 | 2.48342400 | 2.16414200 |
| H | 3.86295600 | 3.05589800 | 3.11129000 |
| H | 2.34926400 | 2.69733200 | -0.66260800 |
| H | 1.37437700 | 4.15465800 | -0.94272100 |
| H | 0.72743300 | 2.84441400 | 0.07487800 |
| H | -0.34256400 | -2.36399600 | -1.23900000 |
| H | -0.70998200 | -1.85654200 | 0.42054900 |
| C | -2.12671400 | -3.27296500 | -0.41390200 |
| H | -1.60344200 | -4.15661800 | -0.04077400 |
| H | -2.92946600 | -3.02441700 | 0.28264600 |
| H | -2.56070900 | -3.51297400 | -1.38676700 |
| H | -2.92124900 | 0.61235900 | 2.58660800 |
| H | -4.14418800 | 1.36762200 | 1.53451000 |
| C | 2.21914700 | -4.28110700 | -0.38057200 |
| H | 2.56601700 | -5.00971200 | -1.11656100 |
| H | 2.73014100 | -4.47269400 | 0.56522500 |
| H | 1.14442900 | -4.41508400 | -0.23686600 |
| C | -4.84564300 | -0.37922300 | 2.59832200 |
| H | -5.67384800 | -0.56689100 | 1.91243200 |
| H | -4.45352500 | -1.33409200 | 2.95460300 |
| H | -5.22289100 | 0.18242000 | 3.45565600 |
| H | -1.92780800 | 3.94316400 | -1.63821300 |

**R = Bu, OH, monoalkylation**

**4**

| | | | |
|---|---|---|---|
| P | 1.90372300 | -0.06675000 | 0.41950500 |
| P | -1.18728600 | 2.35054200 | -0.80527000 |
| O | 1.01064900 | 3.00768400 | 1.82142300 |
| O | -3.29127000 | 0.10652600 | -2.16202700 |
| H | -2.44668500 | -0.40033500 | -2.07888900 |
| O | -0.95237300 | -1.22535300 | -1.95135400 |
| H | -0.41488500 | -1.15148000 | -1.14147300 |
| O | 1.08565100 | -1.18619400 | -0.10815800 |
| O | -0.84089900 | 3.58490000 | -0.04230900 |
| O | 3.41814800 | -0.46316500 | 0.69931600 |
| O | 1.45678300 | 0.58432300 | 1.75526000 |
| O | 1.97554600 | 1.10342200 | -0.69493000 |
| H | 2.70278000 | 1.73529700 | -0.58155600 |
| O | -2.62152200 | 2.40958300 | -1.42155800 |
| H | -2.92598400 | 1.50083000 | -1.81843300 |
| O | -0.24204200 | 2.00855900 | -2.03232200 |
| O | -1.05664400 | 1.02869200 | 0.07642500 |
| C | -4.20267400 | -0.32377700 | -1.13737100 |
| C | -0.05915300 | -1.19642200 | -3.06022500 |
| C | 4.11128000 | -1.33319200 | -0.23438300 |
| C | -1.71937900 | 0.93323300 | 1.36379600 |
| C | 2.17355700 | 3.76636800 | 1.47101200 |
| H | 3.98551700 | -0.93732300 | -1.24707100 |
| H | 2.12142600 | 4.75690500 | 1.92593500 |
| H | 3.04446600 | 3.23613800 | 1.85775200 |
| H | 2.25570400 | 3.86822200 | 0.38577300 |
| H | 0.43326600 | -2.16489200 | -3.18849100 |
| H | -0.64334300 | -0.97405900 | -3.95446900 |
| H | 0.70348300 | -0.42243000 | -2.93560800 |
| H | -3.91174700 | 0.13469700 | -0.18414500 |
| H | -1.13441000 | 1.49556800 | 2.09649800 |
| C | 5.56950300 | -1.37073900 | 0.16135000 |
| H | 5.97720000 | -0.35520400 | 0.12050200 |
| H | 5.64659600 | -1.71603800 | 1.19761600 |
| C | -1.81009100 | -0.53263600 | 1.72755400 |
| H | -2.27639500 | -1.07533500 | 0.89653100 |
| H | -0.79949400 | -0.93365100 | 1.84890600 |
| H | 0.63745300 | 1.68406200 | -1.74892500 |
| C | -4.24780700 | -1.83285800 | -0.99814700 |
| H | -3.22834400 | -2.21342000 | -0.86523300 |
| H | -4.63902800 | -2.27421700 | -1.92129000 |
| H | -2.71451800 | 1.38372900 | 1.28708600 |
| H | -5.18192100 | 0.07299100 | -1.41426800 |
| H | 3.65507800 | -2.32437300 | -0.17918400 |
| C | -5.10465100 | -2.24857600 | 0.19546000 |
| H | -6.12269900 | -1.86539000 | 0.06522400 |
| H | -4.70626400 | -1.77469900 | 1.10064600 |
| C | -5.14183700 | -3.76162800 | 0.38271800 |
| H | -5.73518100 | -4.04122500 | 1.25691200 |
| H | -5.58039700 | -4.25201900 | -0.49122700 |
| H | -4.13317900 | -4.16322100 | 0.51805700 |
| C | -2.61967900 | -0.74966100 | 3.00309100 |
| H | -2.15781600 | -0.19572300 | 3.82769300 |
| H | -3.62570300 | -0.33525100 | 2.87065400 |
| C | -2.71401800 | -2.22879600 | 3.36532800 |
| H | -3.30038600 | -2.38089000 | 4.27469700 |
| H | -3.18776700 | -2.79808100 | 2.55938100 |
| H | -1.71926600 | -2.65218600 | 3.53134100 |
| C | 6.37248600 | -2.29139000 | -0.75505100 |
| H | 5.95112000 | -3.30133100 | -0.71272000 |
| H | 6.27188900 | -1.95053900 | -1.79104600 |
| C | 7.84766500 | -2.33328600 | -0.36889100 |
| H | 7.97213700 | -2.69469400 | 0.65581600 |
| H | 8.41331500 | -2.99513800 | -1.02891300 |
| H | 8.29505900 | -1.33699600 | -0.42950000 |
| H | 1.23720400 | 1.61679700 | 1.76724200 |
| H | 0.26772800 | 3.28638400 | 1.23908700 |

**TS1**

| | | | |
|---|---|---|---|
| P | 1.86778000 | 1.01893500 | 0.55670200 |
| P | -1.73586300 | 0.62223800 | -1.71084700 |
| O | -0.16475000 | 3.75753700 | -0.10836500 |
| O | -1.82028200 | -1.22703900 | -1.85583300 |
| H | -0.79588700 | -1.67249500 | -1.36590600 |
| O | 0.20525300 | -2.03473700 | -0.84609400 |
| H | 0.50340900 | -1.28443800 | -0.21906100 |
| O | 0.92206600 | -0.15094900 | 0.65799000 |
| O | -1.67565200 | 2.24841000 | -1.65920900 |
| O | 3.36596200 | 0.56109200 | 0.91683000 |
| O | 1.58406900 | 2.26921600 | 1.33584000 |
| O | 1.94262300 | 1.37928300 | -1.03718200 |
| H | 2.52142500 | 2.13183300 | -1.22851600 |
| O | -2.91341400 | 0.70477100 | -2.79683000 |
| H | -3.13489500 | -0.13943800 | -3.21046700 |
| O | -0.24825600 | 0.36281100 | -2.24107900 |
| O | -2.09087700 | 0.34112200 | -0.17539000 |
| C | -3.00228300 | -1.93287700 | -1.41376400 |
| C | 1.25410800 | -2.28729900 | -1.79596100 |
| C | 3.85278200 | -0.70014100 | 0.40895700 |
| C | -2.17172800 | 1.32504700 | 0.88079400 |
| C | 0.57853000 | 4.60513900 | -0.97940900 |
| H | 3.69339200 | -0.73443200 | -0.67407400 |
| H | -0.13318500 | 5.18946100 | -1.56330500 |

| | | | |
|---|---|---|---|
| H | 1.21241200 | 5.28749700 | -0.40764000 |
| H | 1.20230000 | 4.02428600 | -1.66644500 |
| H | 2.01838600 | -2.90113300 | -1.31939900 |
| H | 0.81955100 | -2.82929000 | -2.63531200 |
| H | 1.68196000 | -1.34729800 | -2.14710300 |
| H | -3.79607800 | -1.20371600 | -1.23217400 |
| H | -1.19298600 | 1.79343500 | 1.00397900 |
| C | 5.32546100 | -0.81128500 | 0.73696900 |
| H | 5.85924500 | 0.02102800 | 0.26588100 |
| H | 5.45689300 | -0.71544600 | 1.81988300 |
| C | -2.55365700 | 0.58274400 | 2.14299300 |
| H | -3.52669300 | 0.09953300 | 1.99923700 |
| H | -1.81503100 | -0.20645100 | 2.32012900 |
| H | 0.39681700 | 1.06337700 | -2.02748200 |
| C | -2.75205200 | -2.74915500 | -0.15773500 |
| H | -2.30254900 | -2.10326500 | 0.60328800 |
| H | -2.03640000 | -3.54936100 | -0.37439200 |
| H | -2.91088200 | 2.08345100 | 0.61480900 |
| H | -3.31428700 | -2.57769100 | -2.24001600 |
| H | 3.28257700 | -1.50762100 | 0.87755800 |
| C | -4.05141000 | -3.35050200 | 0.37338500 |
| H | -4.51405700 | -3.96828700 | -0.40433000 |
| H | -4.75758700 | -2.54216900 | 0.59305100 |
| C | -3.82586800 | -4.18676800 | 1.62913700 |
| H | -4.76495100 | -4.59673800 | 2.00865000 |
| H | -3.15153600 | -5.02393300 | 1.42702400 |
| H | -3.37840300 | -3.58184500 | 2.42341300 |
| C | -2.60359500 | 1.52285300 | 3.34531800 |
| H | -1.62905900 | 2.00961400 | 3.46097900 |
| H | -3.33344100 | 2.31804100 | 3.15909500 |
| C | -2.96404800 | 0.78554000 | 4.63136900 |
| H | -2.99577200 | 1.46636300 | 5.48521100 |
| H | -3.94439700 | 0.30783300 | 4.54601200 |
| H | -2.22945400 | 0.00554400 | 4.85101700 |
| C | 5.90650500 | -2.14005900 | 0.25772700 |
| H | 5.36802800 | -2.96502700 | 0.73655600 |
| H | 5.74222400 | -2.24112800 | -0.82072100 |
| C | 7.39688800 | -2.25712200 | 0.56129900 |
| H | 7.58328500 | -2.18592500 | 1.63663900 |
| H | 7.79957600 | -3.21176200 | 0.21425100 |
| H | 7.95913800 | -1.45771300 | 0.07033800 |
| H | 0.45980600 | 3.29652700 | 0.49264700 |
| H | -1.07340200 | 2.70236900 | -1.01302700 |

**5**

| | | | |
|---|---|---|---|
| P | -2.03513000 | -0.91430300 | -0.09855500 |
| P | 2.38918700 | -1.28704200 | 0.36968000 |
| O | -0.10763300 | -3.94592000 | -0.30154400 |
| O | 2.89622500 | -0.13954900 | -0.78802500 |
| H | 1.48252200 | 0.68719800 | -1.15772200 |
| O | 0.60011700 | 1.08699600 | -1.35745500 |
| H | -0.36609100 | 0.60956900 | -0.50694600 |
| O | -1.13578300 | 0.31650600 | 0.17587900 |
| O | 1.85353500 | -2.49780400 | 1.42930100 |
| O | -3.53967900 | -0.42775600 | 0.08513200 |
| O | -1.82542500 | -2.12572300 | 0.74243900 |
| O | -1.82092900 | -1.17907000 | -1.66481200 |
| H | -2.27458700 | -1.96470300 | -2.00394200 |
| O | 3.94144200 | -1.73545900 | 0.53494700 |
| O | 1.44918600 | -1.88814900 | -0.77171100 |
| O | 1.85040000 | -0.05664300 | 1.26928900 |
| C | 3.98341400 | 0.75254600 | -0.51725300 |
| C | 0.32109100 | 0.91918100 | -2.75110900 |
| C | -3.99066500 | 0.79225200 | -0.55398100 |
| C | 1.02020800 | -0.13875100 | 2.44542000 |
| C | -0.61350900 | -4.36538600 | -1.56724900 |
| H | -3.67555800 | 0.78076300 | -1.60211900 |
| H | -0.50137800 | -3.57572200 | -2.31715000 |
| H | -0.03724600 | -5.23472900 | -1.88538300 |
| H | -1.66613100 | -4.64931200 | -1.49085100 |
| H | -0.71788800 | 1.20309800 | -2.91687600 |
| H | 0.97145500 | 1.57142500 | -3.33724600 |
| H | 0.46869100 | -0.12027300 | -3.05100700 |
| H | 3.94800800 | 1.08282000 | 0.52629400 |
| H | 0.14125800 | -0.74645400 | 2.22401900 |
| C | -5.49691100 | 0.85433200 | -0.44028400 |
| H | -5.92861300 | -0.02926800 | -0.92185600 |
| H | -5.77510500 | 0.82189500 | 0.61835100 |
| C | 0.62581000 | 1.27602400 | 2.81241600 |
| H | 1.52420900 | 1.84720200 | 3.07069300 |
| H | 0.17321400 | 1.75191100 | 1.93690300 |
| H | 0.92951100 | -2.69597100 | -0.49920900 |
| C | 3.88863800 | 1.94568400 | -1.44965100 |
| H | 2.95817600 | 2.49013300 | -1.25246600 |
| H | 3.84430900 | 1.58467600 | -2.48378800 |
| H | 1.58408300 | -0.60348600 | 3.25940400 |
| H | 4.92504000 | 0.21987900 | -0.67829400 |
| H | -3.51714100 | 1.63772500 | -0.04870400 |
| C | 5.07910000 | 2.88662100 | -1.28059300 |
| H | 6.00552300 | 2.33828800 | -1.48256000 |
| H | 5.13203400 | 3.21973300 | -0.23820000 |
| C | 4.98982300 | 4.09981500 | -2.20139000 |
| H | 5.84750600 | 4.76429800 | -2.07157900 |
| H | 4.96000500 | 3.79142900 | -3.25053500 |
| H | 4.08395400 | 4.67775200 | -1.99673900 |
| C | -0.35884900 | 1.29204200 | 3.97902900 |
| H | -1.25001300 | 0.71729700 | 3.70324200 |
| H | 0.08799400 | 0.78480200 | 4.84129100 |
| C | -0.76230400 | 2.71043500 | 4.36974600 |
| H | -1.46235700 | 2.70913900 | 5.20867400 |
| H | 0.11232100 | 3.29828800 | 4.66306300 |
| H | -1.24356100 | 3.22264900 | 3.53157500 |
| C | -6.05076100 | 2.12289400 | -1.08537700 |
| H | -5.59775400 | 2.99887600 | -0.60896700 |
| H | -5.75715000 | 2.15131600 | -2.14027600 |
| C | -7.56980400 | 2.20526500 | -0.97291800 |
| H | -7.88366100 | 2.19865700 | 0.07485000 |
| H | -7.95355100 | 3.11929800 | -1.43221500 |
| H | -8.04504900 | 1.35456300 | -1.46964600 |
| H | -0.78856100 | -3.40881200 | 0.15184400 |
| H | 2.21399300 | -2.44984700 | 2.32296100 |
| H | 4.05340600 | -2.51308400 | 1.09590500 |

**TS2**

| | | | |
|---|---|---|---|
| P | -1.83506600 | -0.72206300 | -0.16752800 |
| P | 2.14262800 | -1.20149400 | -0.10862800 |
| O | -0.13133100 | -3.51890900 | -1.49499400 |
| O | 2.82109800 | 0.12329500 | -0.76713700 |
| H | 1.48122400 | 1.20890700 | -1.31426100 |
| O | 0.60307900 | 1.56719600 | -1.52564800 |
| H | -0.29041100 | 0.89169700 | -0.59436400 |
| O | -0.86076600 | 0.45197800 | 0.15240400 |

| | | | |
|---|---|---|---|
| O | 1.31416900 | -3.12590100 | 0.56526200 |
| O | -3.31422100 | -0.16523900 | 0.05704700 |
| O | -1.65871700 | -1.95610300 | 0.63270900 |
| O | -1.66188100 | -0.90698200 | -1.75042200 |
| H | -2.20375400 | -1.61335300 | -2.13214800 |
| O | 3.45515900 | -2.10135000 | -0.13424400 |
| O | 1.06080800 | -1.31597700 | -1.22512900 |
| O | 1.75056200 | -0.51647900 | 1.25519700 |
| C | 4.00799100 | 0.73750100 | -0.21973100 |
| C | 0.34366700 | 1.36798100 | -2.91971400 |
| C | -3.72678400 | 1.09195300 | -0.52887000 |
| C | 0.84252800 | -0.92586300 | 2.30688400 |
| C | -1.47023900 | -3.86907300 | -1.83152200 |
| H | -3.55397100 | 1.05264600 | -1.60905700 |
| H | -1.67169200 | -3.47880700 | -2.82956000 |
| H | -1.57890000 | -4.95648800 | -1.85270000 |
| H | -2.18128000 | -3.44555400 | -1.11926400 |
| H | -0.66568900 | 1.72725900 | -3.11767400 |
| H | 1.05506000 | 1.94615900 | -3.51291200 |
| H | 0.41490700 | 0.31055700 | -3.17996600 |
| H | 3.92871300 | 0.77129000 | 0.87048800 |
| H | -0.05404800 | -1.34815200 | 1.85264200 |
| C | -5.19357700 | 1.29257100 | -0.22022600 |
| H | -5.75972700 | 0.44372800 | -0.61800700 |
| H | -5.33024500 | 1.29870500 | 0.86621500 |
| C | 0.52134700 | 0.30579900 | 3.12507300 |
| H | 1.43875400 | 0.68820700 | 3.58540900 |
| H | 0.13536600 | 1.08272600 | 2.45808700 |
| H | 0.49981800 | -2.16296700 | -1.32896000 |
| C | 4.12134600 | 2.13136800 | -0.79978500 |
| H | 3.26787500 | 2.73129800 | -0.46489500 |
| H | 4.06667100 | 2.06321500 | -1.89232900 |
| H | 1.34447100 | -1.67880900 | 2.91814700 |
| H | 4.86980200 | 0.12492500 | -0.49689400 |
| H | -3.11712600 | 1.89182500 | -0.09926300 |
| C | 5.42629300 | 2.80795000 | -0.38726300 |
| H | 6.27136600 | 2.21351500 | -0.75011200 |
| H | 5.49829600 | 2.82269500 | 0.70576300 |
| C | 5.52815900 | 4.23065000 | -0.92810000 |
| H | 6.47812500 | 4.69456300 | -0.65235600 |
| H | 5.45527700 | 4.23816300 | -2.01953800 |
| H | 4.72195300 | 4.85664300 | -0.53535300 |
| C | -0.51381000 | -0.01479200 | 4.20082700 |
| H | -1.43767100 | -0.35278800 | 3.71916900 |
| H | -0.15675400 | -0.84842500 | 4.81569300 |
| C | -0.80467300 | 1.18928200 | 5.09133200 |
| H | -1.60613600 | 0.97462500 | 5.80225300 |
| H | 0.08290200 | 1.47595500 | 5.66263800 |
| H | -1.10995600 | 2.05204600 | 4.49218400 |
| C | -5.71902900 | 2.59562300 | -0.81827000 |
| H | -5.16558400 | 3.43958400 | -0.39291500 |
| H | -5.52570200 | 2.60442500 | -1.89644100 |
| C | -7.21158700 | 2.78056000 | -0.56308400 |
| H | -7.42463400 | 2.79895000 | 0.50952900 |
| H | -7.57540500 | 3.71636900 | -0.99382100 |
| H | -7.78647500 | 1.96123900 | -1.00404900 |
| H | 0.09976000 | -3.72042500 | -0.56248600 |
| H | 1.33980800 | -3.24331700 | 1.51853300 |
| H | 3.19811400 | -2.99275600 | 0.16925300 |

**6**

| | | | |
|---|---|---|---|
| P | 2.10647300 | -0.83929300 | 0.01046600 |
| P | -2.12868600 | -0.94741000 | 0.87672000 |
| O | -0.36498000 | -3.87949000 | -0.25354900 |
| O | -2.46698600 | 0.58796200 | 1.12434700 |
| H | -0.87981900 | 1.62861600 | 0.96878200 |
| O | 0.04795200 | 1.91605700 | 0.97344800 |
| H | 0.87490000 | 1.02418000 | 0.05508500 |
| O | 1.41611100 | 0.44474200 | -0.58553100 |
| O | -2.70544100 | -4.17231900 | 1.09519600 |
| O | 3.67155900 | -0.60803500 | -0.10344800 |
| O | 1.72791000 | -2.10178800 | -0.67791400 |
| O | 1.85281300 | -0.81541200 | 1.58039600 |
| H | 0.89533700 | -0.95305000 | 1.76703900 |
| O | -3.21209900 | -1.74378700 | 1.67753100 |
| O | -0.70913200 | -1.19289800 | 1.23721600 |
| O | -2.48041400 | -1.29129800 | -0.63271300 |
| C | -3.74896100 | 1.17181000 | 0.75615100 |
| C | 0.48899800 | 1.98203700 | 2.33478000 |
| C | 4.29238100 | 0.55456300 | 0.50028400 |
| C | -1.55464000 | -1.01458800 | -1.71839900 |
| C | -0.21330200 | -4.46016600 | -1.54309000 |
| H | 4.26384200 | 0.43157600 | 1.58622500 |
| H | 0.77457400 | -4.91590300 | -1.65488300 |
| H | -0.97255400 | -5.23661400 | -1.64838000 |
| H | -0.35562500 | -3.71660700 | -2.33263300 |
| H | 1.57169500 | 2.10590300 | 2.32126000 |
| H | 0.03225000 | 2.84000700 | 2.83269200 |
| H | 0.23393900 | 1.06552500 | 2.87151200 |
| H | -4.11510900 | 0.67881600 | -0.14834900 |
| H | -0.56509200 | -1.37973100 | -1.43315000 |
| C | 5.71428900 | 0.64455000 | -0.00504400 |
| H | 6.24002600 | -0.28314100 | 0.24374100 |
| H | 5.69983800 | 0.73384500 | -1.09617600 |
| C | -1.52275100 | 0.45871700 | -2.06846300 |
| H | -2.54493200 | 0.81284800 | -2.24447200 |
| H | -1.11699700 | 1.02875900 | -1.22579300 |
| H | 0.27919600 | -3.15239200 | -0.17693100 |
| C | -3.52956800 | 2.64975600 | 0.52615100 |
| H | -2.78355500 | 2.77844600 | -0.26665300 |
| H | -3.11987300 | 3.09525500 | 1.43964100 |
| H | -1.92833600 | -1.61045400 | -2.55168100 |
| H | -4.44396500 | 0.98389600 | 1.57717500 |
| H | 3.71879600 | 1.44426000 | 0.22355600 |
| C | -4.82722300 | 3.35431300 | 0.13803400 |
| H | -5.57155400 | 3.20625200 | 0.92752400 |
| H | -5.23289800 | 2.89240000 | -0.76851600 |
| C | -4.61522200 | 4.84654500 | -0.09647500 |
| H | -5.54780100 | 5.34080000 | -0.37829100 |
| H | -4.23651000 | 5.33298900 | 0.80687000 |
| H | -3.88950900 | 5.01567800 | -0.89718800 |
| C | -0.65931000 | 0.70878800 | -3.30321600 |
| H | 0.31712300 | 0.23257700 | -3.16390500 |
| H | -1.12369000 | 0.23067200 | -4.17198800 |
| C | -0.47064300 | 2.19920700 | -3.56580100 |
| H | 0.12742200 | 2.37486300 | -4.46306400 |
| H | -1.43566400 | 2.69657100 | -3.70213900 |
| H | 0.03740400 | 2.67803600 | -2.72320400 |
| C | 6.44587100 | 1.83697900 | 0.60737900 |
| H | 5.90281200 | 2.75748900 | 0.36770300 |
| H | 6.44032900 | 1.74305800 | 1.69861500 |

| | | | |
|---|---|---|---|
| C | 7.88363600 | 1.94468500 | 0.10932000 |
| H | 7.91236800 | 2.06864800 | -0.97693100 |
| H | 8.39576900 | 2.79892000 | 0.55851500 |
| H | 8.45135600 | 1.04375900 | 0.35881300 |
| H | -1.84748100 | -4.10922600 | 0.61509300 |
| H | -3.32813400 | -4.57150800 | 0.47610400 |
| H | -3.11634900 | -2.74853600 | 1.51107100 |

**R = Et, Et, dialkylation**

**4**

| | | | |
|---|---|---|---|
| P | 2.32817700 | 0.23704600 | -0.24415400 |
| P | -2.14487600 | -0.85199100 | -0.33737600 |
| O | 0.43655000 | -2.86938600 | -1.22484600 |
| H | -0.94414100 | -2.39328700 | -1.35799700 |
| O | -3.03068900 | 1.63725500 | 1.91840800 |
| H | -3.94078200 | 1.82795400 | 2.17277400 |
| O | -0.04506900 | 0.41273800 | 2.30841500 |
| H | 0.81935100 | 0.88149200 | 1.30895400 |
| O | 1.49428700 | 1.25465000 | 0.58319600 |
| O | -1.89833300 | -1.98537600 | -1.39419800 |
| O | 3.51656300 | 1.08285400 | -0.87485800 |
| O | 1.64888200 | -0.50665600 | -1.34414500 |
| H | 0.98443300 | -2.05991800 | -1.34236200 |
| O | 2.94287100 | -0.72205000 | 0.87263500 |
| O | -3.66835000 | -0.46106300 | -0.53287400 |
| O | -1.75615000 | -1.20628600 | 1.05944800 |
| H | -0.75715700 | -0.12712100 | 1.89111000 |
| O | -1.37279700 | 0.45273600 | -0.79294500 |
| C | -2.52474700 | 2.76735200 | 1.20686000 |
| C | 0.62115900 | -0.38773300 | 3.29189000 |
| C | 4.33962300 | 1.93986800 | -0.03996500 |
| C | -1.39308700 | 0.90942800 | -2.17133600 |
| C | 0.67696300 | -3.39335700 | 0.08587100 |
| H | 3.73653700 | 2.79911100 | 0.25967000 |
| H | 4.63507800 | 1.38106100 | 0.85218400 |
| H | -0.20208700 | -3.96683100 | 0.38235400 |
| H | 1.54743000 | -4.05204100 | 0.07311000 |
| H | 0.83445400 | -2.58294900 | 0.80305100 |
| H | 1.50319700 | 0.16191800 | 3.61995800 |
| H | -0.04136800 | -0.55328000 | 4.14326400 |
| H | 0.92750600 | -1.34808500 | 2.87020700 |
| H | -2.45012800 | 3.62782000 | 1.88147600 |
| H | -1.51456700 | 2.49269800 | 0.89506300 |
| C | -3.38562400 | 3.10890000 | 0.00632800 |
| H | -2.98117000 | 3.97786500 | -0.51935000 |
| H | -3.43084900 | 2.26566000 | -0.68741800 |
| H | -4.40450300 | 3.35131500 | 0.32451100 |
| H | -0.94513900 | 0.13300500 | -2.79578500 |
| H | -2.43434400 | 1.05525300 | -2.47088300 |
| C | 5.53994600 | 2.35599800 | -0.85254800 |
| H | 6.17285300 | 3.01235200 | -0.25109300 |
| H | 6.12655100 | 1.48443800 | -1.15049100 |
| H | 5.22955500 | 2.89844400 | -1.74790800 |
| C | -0.61021600 | 2.19673000 | -2.24002600 |
| H | -1.02420000 | 2.93777000 | -1.55336400 |
| H | 0.43868100 | 2.02690400 | -1.99096300 |
| H | -0.66426300 | 2.59461300 | -3.25603100 |
| C | -4.72228100 | -1.29888400 | 0.01086100 |
| H | -4.54400100 | -1.42379200 | 1.08134800 |
| H | -4.67516600 | -2.27460400 | -0.47915400 |
| C | -6.03293200 | -0.60242500 | -0.25412900 |
| H | -6.05206300 | 0.37378900 | 0.23523200 |
| H | -6.18786100 | -0.46668600 | -1.32640000 |
| H | -6.85166100 | -1.20698200 | 0.14196000 |
| C | 3.72771600 | -1.88913600 | 0.51004900 |
| H | 3.52685700 | -2.61880000 | 1.29404300 |
| H | 3.35858700 | -2.28539700 | -0.43933500 |
| C | 5.19791100 | -1.54254100 | 0.44081700 |
| H | 5.39335800 | -0.80947200 | -0.34517600 |
| H | 5.54420000 | -1.14370100 | 1.39668300 |
| H | 5.76860000 | -2.44687300 | 0.21579400 |

**TS1**

| | | | |
|---|---|---|---|
| P | -2.67615600 | 0.27605200 | 0.27111400 |
| P | 2.20279800 | -0.35347900 | -0.30069400 |
| O | -0.54609300 | -1.84673800 | -1.99272800 |
| O | 2.27928700 | 1.60284800 | -0.35900900 |
| H | 1.06275200 | 1.99520900 | -0.53925900 |
| O | -0.01274900 | 2.29518100 | -0.70623900 |
| O | -3.04780600 | -0.62995300 | 1.53342000 |
| O | 2.07221700 | -1.99407300 | -0.35943900 |
| O | -2.55350600 | -0.51452400 | -1.00153200 |
| O | -1.45528100 | 1.06860700 | 0.72486300 |
| H | 0.56893600 | -0.93044000 | -1.71205300 |
| O | -3.94670700 | 1.25497000 | 0.19789800 |
| O | 3.76786600 | -0.22099300 | -0.56684600 |
| O | 1.20413700 | -0.14125700 | -1.49400600 |
| H | -0.65171500 | 1.68683500 | -0.01282700 |
| O | 1.73922400 | -0.15066000 | 1.21486600 |
| C | 2.79848100 | 2.36406600 | 0.73170800 |
| C | -0.25301600 | 3.68396300 | -0.46537800 |
| C | 0.89874600 | -1.09118000 | 1.93864000 |
| C | -0.41731500 | -3.24929900 | -1.75642900 |
| H | -1.33944000 | -3.75973000 | -2.04417000 |
| H | 0.39986400 | -3.62049100 | -2.37433100 |
| H | -0.19654200 | -3.45717700 | -0.70695800 |
| H | 0.38711100 | 4.27212100 | -1.12407600 |
| H | -1.29753300 | 3.90245400 | -0.69488100 |
| H | -0.05012700 | 3.94492300 | 0.57649800 |
| H | 2.77036200 | 3.41227000 | 0.41372900 |
| H | 2.13410500 | 2.26608900 | 1.59780300 |
| C | 4.22052100 | 2.00928800 | 1.12873000 |
| H | 4.59510900 | 2.77859600 | 1.80949400 |
| H | 4.27531900 | 1.04703200 | 1.64109800 |
| H | 4.87251800 | 1.97687200 | 0.25341700 |
| H | 0.03559200 | -1.34095500 | 1.31853100 |
| H | 1.47515300 | -1.99649700 | 2.13723000 |
| C | 0.49006500 | -0.42055100 | 3.22674000 |
| H | 1.37092000 | -0.08651200 | 3.78014900 |
| H | -0.15708700 | 0.43390300 | 3.02782600 |
| H | -0.05665700 | -1.13445600 | 3.84742200 |
| C | 4.69676800 | -1.33581100 | -0.60270900 |
| H | 4.41321900 | -2.00594400 | -1.41468600 |
| H | 4.64509300 | -1.87281300 | 0.34720800 |
| C | 6.07154700 | -0.75177200 | -0.81705100 |
| H | 6.10727200 | -0.18073000 | -1.74691600 |
| H | 6.35043800 | -0.09963200 | 0.01268700 |
| H | 6.80040800 | -1.56300700 | -0.88064900 |
| C | -4.29494000 | 1.92017000 | -1.03975200 |
| H | -4.66986000 | 2.90051900 | -0.74500900 |
| H | -3.39481400 | 2.06299500 | -1.64373100 |
| C | -5.34495600 | 1.13124700 | -1.78826100 |

| | | | |
|---|---|---|---|
| H | -4.95690700 | 0.15839900 | -2.09599500 |
| H | -6.22774600 | 0.98192000 | -1.16231700 |
| H | -5.64395200 | 1.68273800 | -2.68314800 |
| H | -1.27287500 | -1.47547700 | -1.43482600 |
| C | -4.15467800 | -1.56112500 | 1.44220000 |
| H | -4.63785300 | -1.53494700 | 2.41896700 |
| H | -4.87009400 | -1.20210900 | 0.69683500 |
| C | -3.65180500 | -2.94656900 | 1.10600600 |
| H | -2.92389400 | -3.28088900 | 1.84873700 |
| H | -4.48962800 | -3.64816600 | 1.10426400 |
| H | -3.18513900 | -2.96369100 | 0.11855800 |
| H | 2.49685800 | -2.48965400 | 0.35242300 |

**5**

| | | | |
|---|---|---|---|
| P | 1.92429900 | -0.59857100 | 0.37090000 |
| P | -2.29238900 | 0.36093100 | -0.12496200 |
| O | 0.08600800 | 2.60069000 | 1.67002600 |
| H | -1.38982400 | 2.14494600 | 0.92740600 |
| O | -2.43264700 | -1.25959200 | -0.87059700 |
| H | -1.24943300 | -1.70067000 | -1.49910000 |
| O | -0.37336600 | -2.14424400 | -1.88223700 |
| H | 0.38782800 | -2.01780700 | -1.11002200 |
| O | 1.31075300 | -1.87875900 | -0.17606900 |
| O | -2.23829600 | 1.88190400 | 0.49997700 |
| O | 3.36186300 | -0.96381000 | 0.99382700 |
| O | 1.19166000 | 0.18423700 | 1.41715700 |
| H | 0.64945200 | 1.80655100 | 1.54681400 |
| O | 2.24280500 | 0.30097200 | -0.92633700 |
| O | -3.80365000 | 0.67656600 | -0.60498300 |
| H | -4.16221500 | -0.03489300 | -1.15045400 |
| O | -1.08739400 | 0.52841900 | -1.18559500 |
| O | -1.98455100 | -0.41582300 | 1.24416300 |
| C | -2.97269800 | -2.41100300 | -0.19265700 |
| C | 0.04515500 | -1.51495700 | -3.10878700 |
| C | 4.25125300 | -1.84285400 | 0.27008300 |
| C | -2.04890800 | 0.13254000 | 2.57949800 |
| C | 0.79740000 | 3.79051700 | 1.35470800 |
| H | 3.82143700 | -2.84714000 | 0.27193600 |
| H | 4.33082300 | -1.49724500 | -0.76534800 |
| H | 0.13841000 | 4.63355400 | 1.56566400 |
| H | 1.69508400 | 3.88404100 | 1.97243600 |
| H | 1.08519000 | 3.82517100 | 0.29924300 |
| H | 0.56554800 | -2.26036300 | -3.70813400 |
| H | -0.84142800 | -1.16484100 | -3.63587100 |
| H | 0.70824100 | -0.67861200 | -2.88586900 |
| H | -2.96529100 | -3.20325300 | -0.94545900 |
| H | -2.29603900 | -2.69783300 | 0.61499400 |
| C | -4.38202500 | -2.21275400 | 0.32932500 |
| H | -4.72315000 | -3.15051100 | 0.77491600 |
| H | -4.42943900 | -1.43764500 | 1.09714300 |
| H | -5.07282200 | -1.95715300 | -0.47900600 |
| H | -1.25945400 | 0.87320900 | 2.70604600 |
| H | -3.01814600 | 0.61584900 | 2.71753100 |
| C | 5.59257400 | -1.81584900 | 0.96165000 |
| H | 6.28821500 | -2.47558800 | 0.43825700 |
| H | 6.00646600 | -0.80516000 | 0.96369300 |
| H | 5.49973200 | -2.16067000 | 1.99375500 |
| C | -1.86568400 | -1.02731800 | 3.52850700 |
| H | -2.66046100 | -1.76592800 | 3.39989200 |
| H | -0.90088400 | -1.51137500 | 3.36062300 |
| H | -1.89534500 | -0.66246000 | 4.55781100 |
| C | -0.55907200 | 1.78414500 | -1.66653000 |
| H | 0.31452000 | 1.49038500 | -2.24764400 |
| H | -0.22274100 | 2.39118900 | -0.82553800 |
| C | -1.56062100 | 2.51600000 | -2.53258000 |
| H | -1.89334500 | 1.87621700 | -3.35383300 |
| H | -2.43061200 | 2.83416800 | -1.95509600 |
| H | -1.08676400 | 3.40349700 | -2.95949100 |
| C | 2.87215100 | 1.59415100 | -0.77589200 |
| H | 2.36131800 | 2.25769400 | -1.47517100 |
| H | 2.70277100 | 1.96728200 | 0.23793400 |
| C | 4.34965000 | 1.50910600 | -1.09102400 |
| H | 4.87174500 | 0.88018500 | -0.36680300 |
| H | 4.50546500 | 1.10458500 | -2.09365800 |
| H | 4.78595800 | 2.51040200 | -1.05110600 |

**TS2**

| | | | |
|---|---|---|---|
| P | -1.78018100 | -0.56294200 | -0.34507200 |
| P | 2.28677300 | 0.27652700 | 0.07295100 |
| O | -0.58338400 | 3.05172300 | -1.69643000 |
| H | 1.07884100 | 2.37002400 | -1.15806000 |
| O | 2.79417700 | -0.90691500 | 1.11242600 |
| H | 1.31867400 | -1.56009200 | 1.87474100 |
| O | 0.46889400 | -2.00458900 | 2.07087700 |
| H | -0.35563100 | -1.84216600 | 0.90930800 |
| O | -0.96512000 | -1.82453700 | 0.05767300 |
| O | 1.83782200 | 1.76243500 | -1.18492800 |
| O | -3.13331800 | -1.12286800 | -0.97512200 |
| O | -1.16773500 | 0.38483400 | -1.31494900 |
| H | -1.00485300 | 2.18135300 | -1.56876000 |
| O | -2.14695700 | 0.10747400 | 1.05762000 |
| O | 3.58546400 | 1.14270100 | -0.01125400 |
| O | 1.12763300 | 0.66858700 | 1.09172800 |
| O | 1.87462900 | -0.75802000 | -1.05814200 |
| C | 3.54057600 | -2.06194100 | 0.67640900 |
| C | -0.09349600 | -1.42862000 | 3.25490100 |
| C | -3.89932100 | -2.14463600 | -0.28665100 |
| C | 1.75273000 | -0.57796100 | -2.48851600 |
| C | -1.34560600 | 4.06582400 | -1.05554300 |
| H | -3.34623700 | -3.08377700 | -0.35141000 |
| H | -4.00031300 | -1.86242300 | 0.76535100 |
| H | -0.87153400 | 5.02257300 | -1.27733900 |
| H | -2.37210400 | 4.08807800 | -1.43301400 |
| H | -1.36744500 | 3.93335900 | 0.03174100 |
| H | -1.10389500 | -1.82134500 | 3.36469600 |
| H | 0.50114900 | -1.71643800 | 4.12390800 |
| H | -0.12710100 | -0.34009400 | 3.17582700 |
| H | 3.77202800 | -2.59541200 | 1.59940100 |
| H | 2.88591700 | -2.69049900 | 0.06821600 |
| C | 4.81336500 | -1.72315500 | -0.07108100 |
| H | 5.35422200 | -2.65204400 | -0.26987900 |
| H | 4.60790400 | -1.24101900 | -1.02986500 |
| H | 5.45582100 | -1.07055300 | 0.52387600 |
| H | 0.92713700 | 0.10005400 | -2.68974900 |
| H | 2.68040100 | -0.14009600 | -2.86191800 |
| C | -5.24467700 | -2.24639700 | -0.96103900 |
| H | -5.83156400 | -3.03039000 | -0.47730400 |
| H | -5.79151200 | -1.30446800 | -0.88402900 |
| H | -5.12707800 | -2.50226000 | -2.01597500 |
| C | 1.50462500 | -1.94884400 | -3.07011000 |
| H | 2.33209400 | -2.62640800 | -2.84751600 |
| H | 0.58021100 | -2.37213300 | -2.66981400 |

| | | | |
|---|---|---|---|
| H | 1.40595000 | -1.86593500 | -4.15515000 |
| C | 0.43738000 | 1.91758900 | 1.31140200 |
| H | -0.21666700 | 1.69233400 | 2.15354200 |
| H | -0.18601600 | 2.12743000 | 0.44519200 |
| C | 1.36504400 | 3.06072700 | 1.65992300 |
| H | 2.02180900 | 2.77887700 | 2.48643000 |
| H | 1.97582800 | 3.37664700 | 0.81303300 |
| H | 0.76109600 | 3.91345600 | 1.98044100 |
| C | -2.88935600 | 1.35463300 | 1.09827500 |
| H | -2.54462400 | 1.86028900 | 1.99997000 |
| H | -2.61570600 | 1.95927400 | 0.22929800 |
| C | -4.37998400 | 1.09578300 | 1.14265900 |
| H | -4.71986800 | 0.60188800 | 0.22985700 |
| H | -4.63683800 | 0.47842700 | 2.00589900 |
| H | -4.90455900 | 2.05018400 | 1.23327500 |
| H | 2.96105800 | 1.89489700 | -0.70218300 |

**6**

| | | | |
|---|---|---|---|
| P | -1.85554500 | -0.10369700 | -0.64383300 |
| P | 2.26524200 | -0.24361500 | 0.04787700 |
| O | 0.18481200 | 3.21751800 | -0.40233000 |
| H | 1.99166700 | 3.31166500 | -0.33464700 |
| O | 2.40041800 | -1.73586700 | 0.57915500 |
| H | 0.74009800 | -2.30201800 | 1.41562000 |
| O | -0.18771700 | -2.57336100 | 1.32507600 |
| H | -0.72719400 | -1.88739400 | 0.13871000 |
| O | -1.10701400 | -1.46933300 | -0.72675000 |
| O | 2.95981600 | 3.38536600 | -0.25458300 |
| O | -3.27527200 | -0.34572500 | -1.32864200 |
| O | -1.22327800 | 1.06765100 | -1.30729300 |
| H | -0.34469300 | 2.44020100 | -0.66543500 |
| O | -2.11068000 | 0.07828800 | 0.91760800 |
| O | 3.45452800 | 0.62479300 | 0.24439400 |
| O | 0.96359200 | 0.25933700 | 0.79685700 |
| O | 1.82545100 | -0.45049200 | -1.45480900 |
| C | 3.09758800 | -2.78160400 | -0.16258600 |
| C | -0.89978800 | -2.35016500 | 2.54777600 |
| C | -4.04905700 | -1.53011400 | -1.00737900 |
| C | 1.85925600 | 0.67974700 | -2.37575400 |
| C | -0.52776000 | 3.98263700 | 0.55982200 |
| H | -3.55910400 | -2.38856100 | -1.47077500 |
| H | -4.05389200 | -1.66685100 | 0.07806400 |
| H | 0.10231700 | 4.82857500 | 0.83969600 |
| H | -1.46373200 | 4.36881200 | 0.14413000 |
| H | -0.74929800 | 3.39993100 | 1.45905300 |
| H | -1.91742900 | -2.70814800 | 2.39303700 |
| H | -0.43567800 | -2.91688700 | 3.35688900 |
| H | -0.92574400 | -1.28793900 | 2.79803700 |
| H | 3.04969000 | -3.64528500 | 0.49843000 |
| H | 2.51731700 | -2.98824400 | -1.06331900 |
| C | 4.52615700 | -2.40707900 | -0.48184400 |
| H | 5.01677500 | -3.27065300 | -0.93668600 |
| H | 4.57811200 | -1.57754200 | -1.19115500 |
| H | 5.06912100 | -2.13584500 | 0.42587400 |
| H | 1.31085700 | 1.50923800 | -1.92418700 |
| H | 2.90366300 | 0.96708900 | -2.51418500 |
| C | -5.44386200 | -1.32844200 | -1.54450100 |
| H | -6.04516300 | -2.21497000 | -1.33087100 |
| H | -5.91892300 | -0.46403400 | -1.07588700 |
| H | -5.42012700 | -1.17687300 | -2.62557700 |
| C | 1.22279300 | 0.22839200 | -3.66444800 |
| H | 1.77909400 | -0.60592200 | -4.09707000 |
| H | 0.18900000 | -0.07916100 | -3.49584600 |
| H | 1.22904300 | 1.05667300 | -4.37637400 |
| C | 1.03920300 | 1.07984900 | 1.99586300 |
| H | -0.00228300 | 1.30726300 | 2.22112500 |
| H | 1.56036000 | 2.00615000 | 1.74391300 |
| C | 1.70803200 | 0.34920600 | 3.13912600 |
| H | 1.20711100 | -0.59890800 | 3.34658900 |
| H | 2.76258800 | 0.15512100 | 2.92831900 |
| H | 1.65414300 | 0.97126900 | 4.03585900 |
| C | -2.84441200 | 1.22688300 | 1.42044700 |
| H | -2.32381100 | 1.52385700 | 2.33112500 |
| H | -2.77440000 | 2.04397600 | 0.69760700 |
| C | -4.27996500 | 0.85016700 | 1.70654900 |
| H | -4.81423900 | 0.59802000 | 0.78801500 |
| H | -4.32176100 | -0.00012500 | 2.39112300 |
| H | -4.78600300 | 1.69715300 | 2.17631700 |
| H | 3.25070000 | 2.47778000 | -0.07408200 |

**R = Et, OH, monoalkylation**

**4**

| | | | |
|---|---|---|---|
| P | -2.01660300 | -0.06968300 | -0.34486300 |
| P | 1.88435000 | 0.43326400 | 0.07063300 |
| O | 0.35192600 | 3.46101500 | 0.15085600 |
| H | 0.44823400 | 1.48309600 | -1.73243900 |
| O | 2.31928500 | -0.58156400 | 1.29317100 |
| H | 0.79665200 | -0.87144500 | 2.30349500 |
| O | -0.10319600 | -1.18691600 | 2.50661500 |
| H | -0.82614000 | -1.19428300 | 1.24494200 |
| O | -1.36201100 | -1.29567800 | 0.35998000 |
| O | 1.31645000 | 1.82538200 | -1.47023300 |
| O | -3.41641500 | -0.60959500 | -0.88032200 |
| O | -1.29055500 | 0.56990700 | -1.46971700 |
| H | 0.79881500 | 3.02036300 | -0.65512900 |
| O | -2.33852400 | 0.91338900 | 0.87909900 |
| H | -2.62284600 | 1.80092600 | 0.61453900 |
| O | 3.27851100 | 1.18039400 | -0.14621000 |
| H | 3.93566800 | 0.92897600 | 0.52136500 |
| O | 0.78533100 | 1.11487600 | 0.95721800 |
| O | 1.51067000 | -0.71694300 | -0.94001700 |
| C | 2.92529800 | -1.87718100 | 1.07505600 |
| C | -0.69597800 | -0.34233800 | 3.49920400 |
| C | -4.29311300 | -1.37358900 | -0.01370600 |
| C | 1.71083100 | -0.76878900 | -2.37616500 |
| C | -0.93801400 | 3.93323900 | -0.22287200 |
| H | -3.84275400 | -2.35593900 | 0.14110400 |
| H | -4.37152900 | -0.86106400 | 0.94881900 |
| H | -1.52679900 | 4.08689100 | 0.68321200 |
| H | -0.85576000 | 4.88591900 | -0.75315700 |
| H | -1.44818400 | 3.21207000 | -0.86995300 |
| H | -1.74722700 | -0.61958000 | 3.57418200 |
| H | -0.20974700 | -0.50415200 | 4.46287300 |
| H | -0.61376700 | 0.70797800 | 3.21360600 |
| H | 3.06188900 | -2.27900800 | 2.07937200 |
| H | 2.21218600 | -2.50747100 | 0.54040800 |
| C | 4.25062100 | -1.80358600 | 0.34586000 |
| H | 4.67303500 | -2.80938900 | 0.28443700 |
| H | 4.13862100 | -1.42462900 | -0.67335900 |
| H | 4.95924400 | -1.16962500 | 0.88587100 |
| H | 0.84857500 | -0.30766200 | -2.85547500 |
| H | 2.60999000 | -0.20483300 | -2.62641200 |

| | | | |
|---|---|---|---|
| C | -5.63513600 | -1.47719100 | -0.69447500 |
| H | -6.31012300 | -2.07254300 | -0.07565700 |
| H | -6.07410000 | -0.48769600 | -0.83699800 |
| H | -5.53481500 | -1.96510100 | -1.66625900 |
| C | 1.83794700 | -2.22865500 | -2.73744400 |
| H | 2.69908800 | -2.68098400 | -2.24036300 |
| H | 0.93582200 | -2.77520900 | -2.45390600 |
| H | 1.97260700 | -2.31949000 | -3.81785900 |
| H | 0.49066300 | 2.06458200 | 0.69592900 |

**TS1**

| | | | |
|---|---|---|---|
| P | 2.51676600 | -0.48214200 | 0.28248200 |
| P | -2.05074200 | -0.11928800 | -0.83809400 |
| O | 0.82545300 | 0.37579000 | -2.97015200 |
| O | -2.23189200 | -1.84110500 | 0.03053300 |
| H | -0.92732700 | -2.56869900 | -0.08708900 |
| O | 0.00925400 | -2.99873100 | -0.05701200 |
| O | 2.50973200 | 0.94384900 | 0.97885500 |
| O | -1.81509200 | 1.27692900 | -1.71029500 |
| O | 2.66913900 | -0.46974600 | -1.20671200 |
| O | 1.21485000 | -1.10807800 | 0.83362800 |
| H | -0.39782100 | -0.31392400 | -2.28203800 |
| O | 3.77075800 | -1.18196200 | 0.98572500 |
| O | -3.61994900 | -0.28021400 | -1.11188900 |
| O | -1.00635200 | -0.90410800 | -1.72938400 |
| H | 0.72895800 | -1.98329300 | 0.35765900 |
| O | -1.63445000 | 0.45833800 | 0.59547200 |
| C | -2.67332400 | -1.94302100 | 1.37819400 |
| C | 0.03308700 | -4.04803800 | 0.91500600 |
| C | -0.79965600 | 1.62661000 | 0.79223100 |
| C | 0.87663000 | 1.79625200 | -3.08370900 |
| H | 1.82057600 | 2.10616600 | -3.54031600 |
| H | 0.05308300 | 2.10804000 | -3.72532100 |
| H | 0.76801300 | 2.27618700 | -2.10728200 |
| H | -0.75218900 | -4.77346800 | 0.69565100 |
| H | 1.00336300 | -4.54176900 | 0.85314800 |
| H | -0.10584200 | -3.64921300 | 1.92368500 |
| H | -2.68657700 | -3.01636200 | 1.60607600 |
| H | -1.94448100 | -1.47427400 | 2.05250400 |
| C | -4.05927200 | -1.37414200 | 1.64920400 |
| H | -4.06188000 | -0.29263600 | 1.47720200 |
| H | -4.77377400 | -1.81426900 | 0.94475200 |
| H | 0.06164300 | 1.56672300 | 0.12002100 |
| H | -1.37804100 | 2.52447300 | 0.55630500 |
| C | -0.35500900 | 1.63679300 | 2.23721700 |
| H | -1.23617300 | 1.59692800 | 2.88710700 |
| H | 0.24160400 | 0.74046500 | 2.42942700 |
| C | -4.48909300 | 0.76011700 | -1.61404200 |
| H | -4.17721400 | 1.03923800 | -2.62221400 |
| H | -4.42054100 | 1.63376500 | -0.95835400 |
| C | -5.89621600 | 0.20247300 | -1.61069500 |
| H | -5.91753500 | -0.70663600 | -2.22097500 |
| H | -6.16677100 | -0.08188300 | -0.58823200 |
| C | 4.41313700 | -2.33405600 | 0.38615500 |
| H | 4.68367900 | -2.98169900 | 1.22142600 |
| H | 3.69861500 | -2.86810500 | -0.24781600 |
| C | 5.63833400 | -1.91237600 | -0.39809600 |
| H | 5.33429300 | -1.24328000 | -1.20979200 |
| H | 6.30222800 | -1.34653400 | 0.26491600 |
| H | 1.55029100 | 0.08034900 | -2.37658500 |
| C | 3.59008200 | 1.88649300 | 0.77180600 |
| H | 3.63919400 | 2.45652300 | 1.69988900 |
| H | 4.53265800 | 1.34257300 | 0.64901600 |
| C | 3.30799800 | 2.77465500 | -0.42507200 |
| H | 2.33780300 | 3.26433500 | -0.28089600 |
| H | 3.23016700 | 2.14547800 | -1.31620200 |
| H | -2.24503900 | 2.07175800 | -1.37269900 |
| C | 4.39543500 | 3.83115900 | -0.62941500 |
| H | 5.36171000 | 3.33529900 | -0.77352300 |
| H | 4.18053600 | 4.36733300 | -1.55808000 |
| C | 4.49479800 | 4.83459800 | 0.51918300 |
| H | 4.83908900 | 4.36534600 | 1.44407300 |
| H | 5.19772900 | 5.63549900 | 0.27751700 |
| H | 3.52062000 | 5.29298300 | 0.71771500 |
| C | 6.37867600 | -3.12041400 | -0.96723700 |
| H | 5.70061100 | -3.69194100 | -1.61024200 |
| H | 6.66878500 | -3.78541600 | -0.14644500 |
| C | 7.61679400 | -2.71370000 | -1.76057700 |
| H | 7.34565500 | -2.07663100 | -2.60726700 |
| H | 8.31650700 | -2.15533800 | -1.13207600 |
| H | 8.14186600 | -3.58769300 | -2.15340800 |
| C | -4.50029700 | -1.65982400 | 3.08287700 |
| H | -4.50768800 | -2.74244900 | 3.25178000 |
| H | -3.76476400 | -1.24082400 | 3.77915500 |
| C | -5.88039700 | -1.08548000 | 3.39010500 |
| H | -5.88778900 | 0.00123800 | 3.26374900 |
| H | -6.63457500 | -1.50418700 | 2.71695400 |
| H | -6.18727000 | -1.30555000 | 4.41576600 |
| C | -6.90155900 | 1.21667600 | -2.15152200 |
| H | -6.86227200 | 2.12771500 | -1.54454300 |
| H | -6.61586100 | 1.50341900 | -3.16926200 |
| C | -8.32313000 | 0.66274900 | -2.15406500 |
| H | -8.38830600 | -0.23635800 | -2.77358700 |
| H | -8.63881300 | 0.39472200 | -1.14173100 |
| H | -9.03481700 | 1.39331600 | -2.54597000 |
| C | 0.46690900 | 2.88812300 | 2.54508500 |
| H | -0.18262700 | 3.76928200 | 2.51020400 |
| H | 1.22459400 | 3.02399800 | 1.76620400 |
| C | 1.15133000 | 2.80005500 | 3.90531600 |
| H | 0.41697300 | 2.66265500 | 4.70474100 |
| H | 1.83986400 | 1.95032600 | 3.93573000 |
| H | 1.72177500 | 3.70584200 | 4.12589900 |

**5**

| | | | |
|---|---|---|---|
| P | 2.51181200 | -0.47514500 | 0.25092400 |
| P | -2.06678700 | -0.21378600 | -0.74239300 |
| O | 0.80869300 | 0.41401600 | -3.03868000 |
| O | -2.22381100 | -1.73463500 | 0.02604900 |
| H | -0.80307100 | -2.68326600 | -0.24610000 |
| O | 0.08885900 | -3.09556200 | -0.16039800 |
| O | 2.46706300 | 0.94232500 | 0.95396600 |
| O | -1.81017300 | 1.20445600 | -1.63890200 |
| O | 2.64221400 | -0.47116600 | -1.23730800 |
| O | 1.21658200 | -1.12964600 | 0.81636900 |
| H | -0.47108800 | -0.32390500 | -2.26415100 |
| O | 3.77301900 | -1.15455000 | 0.95347200 |
| O | -3.64988900 | -0.28941100 | -1.07039000 |
| O | -0.99217400 | -0.94070500 | -1.68451300 |
| H | 0.80530400 | -1.96559200 | 0.33015900 |
| O | -1.64754700 | 0.45333200 | 0.66846400 |
| C | -2.68550300 | -1.92770900 | 1.36544800 |
| C | 0.04660700 | -4.11440200 | 0.84475800 |

| | | | |
|---|---|---|---|
| C | -0.83935500 | 1.63495300 | 0.84102800 |
| C | 0.86277900 | 1.83603200 | -3.09648900 |
| H | 1.80350600 | 2.16822800 | -3.54543600 |
| H | 0.03512000 | 2.17423900 | -3.71996200 |
| H | 0.75892200 | 2.27856100 | -2.10169500 |
| H | -0.73601300 | -4.83887500 | 0.61335100 |
| H | 1.01345400 | -4.61740800 | 0.84156700 |
| H | -0.13123500 | -3.68030900 | 1.83236100 |
| H | -2.68455400 | -3.01315200 | 1.50555400 |
| H | -1.95867700 | -1.49787200 | 2.06293100 |
| C | -4.07706600 | -1.39146300 | 1.66513900 |
| H | -4.09706200 | -0.30305900 | 1.54726200 |
| H | -4.79096100 | -1.80915200 | 0.94756100 |
| H | 0.00223900 | 1.60232500 | 0.14310800 |
| H | -1.44273200 | 2.52314200 | 0.62937500 |
| C | -0.34323300 | 1.65070300 | 2.27056700 |
| H | -1.19885600 | 1.60029100 | 2.95314700 |
| H | 0.26897100 | 0.75947700 | 2.43808800 |
| C | -4.47209200 | 0.77309000 | -1.58352900 |
| H | -4.12244400 | 1.06585400 | -2.57547300 |
| H | -4.40961600 | 1.63601200 | -0.91153200 |
| C | -5.89443000 | 0.25510500 | -1.64143300 |
| H | -5.91998600 | -0.63705900 | -2.27646100 |
| H | -6.20743700 | -0.04929300 | -0.63686600 |
| C | 4.42518200 | -2.30888600 | 0.36599800 |
| H | 4.70209000 | -2.94287700 | 1.20941800 |
| H | 3.71413900 | -2.85648900 | -0.26011100 |
| C | 5.64503100 | -1.88456500 | -0.42445400 |
| H | 5.33343400 | -1.23384800 | -1.24820500 |
| H | 6.30062700 | -1.29877600 | 0.22930700 |
| H | 1.52317900 | 0.10090800 | -2.44738400 |
| C | 3.54307600 | 1.90050000 | 0.78656000 |
| H | 3.55441600 | 2.46408900 | 1.71939400 |
| H | 4.49476100 | 1.36762500 | 0.69120800 |
| C | 3.28738100 | 2.79184900 | -0.41346100 |
| H | 2.31104100 | 3.27539800 | -0.29294200 |
| H | 3.23686200 | 2.16698800 | -1.30977200 |
| H | -2.25166100 | 1.99471400 | -1.30865100 |
| C | 4.37413600 | 3.85531300 | -0.58194200 |
| H | 5.34587300 | 3.36544500 | -0.70873100 |
| H | 4.17771000 | 4.39938800 | -1.51005600 |
| C | 4.44185000 | 4.84718600 | 0.57888900 |
| H | 4.76620800 | 4.36952500 | 1.50675800 |
| H | 5.14713000 | 5.65318400 | 0.36231300 |
| H | 3.46133400 | 5.29958700 | 0.75921900 |
| C | 6.40146600 | -3.09318100 | -0.97083200 |
| H | 5.73236500 | -3.68418100 | -1.60553000 |
| H | 6.69728700 | -3.74001000 | -0.13764800 |
| C | 7.63690200 | -2.68498900 | -1.76750700 |
| H | 7.36060300 | -2.06783300 | -2.62718200 |
| H | 8.32642000 | -2.10535200 | -1.14703500 |
| H | 8.17522500 | -3.55926500 | -2.14131000 |
| C | -4.49609000 | -1.75646900 | 3.08771300 |
| H | -4.47199100 | -2.84558800 | 3.20509500 |
| H | -3.76630300 | -1.34925600 | 3.79657600 |
| C | -5.88887100 | -1.23556000 | 3.43037400 |
| H | -5.92758800 | -0.14498600 | 3.35282800 |
| H | -6.63693600 | -1.64539400 | 2.74523600 |
| H | -6.17981400 | -1.50951400 | 4.44755800 |
| C | -6.85376600 | 1.31017400 | -2.18708600 |

| | | | |
|---|---|---|---|
| H | -6.81686000 | 2.19985100 | -1.54895400 |
| H | -6.52046900 | 1.62337700 | -3.18236500 |
| C | -8.28724800 | 0.79371500 | -2.26484600 |
| H | -8.35071900 | -0.07882200 | -2.92148000 |
| H | -8.64807700 | 0.49477200 | -1.27649400 |
| H | -8.96605400 | 1.55646000 | -2.65374400 |
| C | 0.47967000 | 2.90710400 | 2.55160200 |
| H | -0.17685000 | 3.78376700 | 2.54508400 |
| H | 1.20474100 | 3.05168800 | 1.74374500 |
| C | 1.21891900 | 2.82030300 | 3.88293500 |
| H | 0.51851600 | 2.67455600 | 4.71086500 |
| H | 1.91438800 | 1.97551400 | 3.88250800 |
| H | 1.79104000 | 3.72967700 | 4.08410000 |

**TS2**

| | | | |
|---|---|---|---|
| P | -2.56502200 | 0.15376400 | -0.78531600 |
| P | 2.34764100 | -0.16600900 | -0.96718500 |
| O | 0.36911600 | -2.65817100 | -2.57678900 |
| O | 2.49813100 | 1.43967400 | -1.26186300 |
| H | 0.93926600 | 2.02619400 | -2.06401600 |
| O | -0.00601500 | 2.19645000 | -2.23363000 |
| O | -2.54495500 | -0.98922000 | 0.31753700 |
| O | 2.07713400 | -2.33331000 | -0.79724700 |
| O | -3.57035600 | -0.03049800 | -1.86403800 |
| O | -1.05361500 | 0.14282800 | -1.18938900 |
| H | 1.04196200 | -1.25258300 | -2.43986400 |
| O | -2.78338700 | 1.51275700 | 0.03116800 |
| O | 3.90343200 | -0.46241300 | -0.93339200 |
| O | 1.56196900 | -0.39143400 | -2.30354800 |
| H | -0.67463900 | 0.97117900 | -1.65741800 |
| O | 1.62694600 | -0.00479400 | 0.42592900 |
| C | 2.82628000 | 2.40908400 | -0.24216100 |
| C | -0.41030400 | 3.36728800 | -1.51419200 |
| C | 0.78514000 | -0.89602700 | 1.19891800 |
| C | -0.97700800 | -2.86436100 | -2.15404700 |
| H | -1.11081100 | -2.55906000 | -1.11328900 |
| H | -1.63637200 | -2.26767500 | -2.78669000 |
| H | -1.24669300 | -3.92002400 | -2.25505400 |
| H | 0.20082500 | 4.22234400 | -1.80889400 |
| H | -1.44982200 | 3.56400400 | -1.77590300 |
| H | -0.33077400 | 3.20951300 | -0.43488600 |
| H | 2.86406600 | 3.35820600 | -0.78093200 |
| H | 2.00170900 | 2.44966000 | 0.47521300 |
| C | 4.14457200 | 2.15824800 | 0.46547000 |
| H | 4.08871600 | 1.23517700 | 1.05260800 |
| H | 4.93411200 | 2.02170500 | -0.28052000 |
| H | 0.16327500 | -1.47406200 | 0.51826000 |
| H | 1.43243600 | -1.56646400 | 1.76907700 |
| C | -0.06041000 | -0.03221800 | 2.10723300 |
| H | 0.58584300 | 0.61543600 | 2.70938100 |
| H | -0.68885900 | 0.61503500 | 1.48703600 |
| C | 4.59492900 | -1.44576800 | -0.12703900 |
| H | 4.54407900 | -2.40609400 | -0.63855600 |
| H | 4.09399100 | -1.52661600 | 0.84235100 |
| C | 6.02736700 | -0.98369200 | 0.03672900 |
| H | 6.46778800 | -0.83013700 | -0.95431700 |
| H | 6.04678500 | -0.02229700 | 0.55876500 |
| C | -3.81156200 | 2.47211700 | -0.31385900 |
| H | -3.44296600 | 3.42494800 | 0.06955200 |
| H | -3.89329300 | 2.53833600 | -1.40142700 |
| C | -5.13939700 | 2.10755200 | 0.31696500 |

| | | | |
|---|---|---|---|
| H | -5.47926100 | 1.14622800 | -0.08466100 |
| H | -4.99683800 | 1.98362300 | 1.39639700 |
| H | 0.98660500 | -2.84246200 | -1.80625400 |
| C | -3.77076800 | -1.62608200 | 0.74934900 |
| H | -3.66709900 | -1.74465200 | 1.82855000 |
| H | -4.62354800 | -0.96842300 | 0.55241800 |
| C | -3.93103800 | -2.95847200 | 0.04190800 |
| H | -3.07802600 | -3.59656800 | 0.29992000 |
| H | -3.89446600 | -2.78248000 | -1.03815000 |
| H | 2.30173300 | -2.69888700 | 0.06234800 |
| C | -5.23945600 | -3.65958800 | 0.41084100 |
| H | -6.08372800 | -3.02663400 | 0.11625100 |
| H | -5.31605000 | -4.57616700 | -0.18069700 |
| C | -5.34796800 | -4.00629600 | 1.89509800 |
| H | -5.40239800 | -3.11115300 | 2.52003900 |
| H | -6.24466200 | -4.59847700 | 2.09312700 |
| H | -4.48168900 | -4.59076900 | 2.22072300 |
| C | -6.19420300 | 3.17943900 | 0.04956200 |
| H | -6.31447700 | 3.30714400 | -1.03172800 |
| H | -5.84151500 | 4.13923800 | 0.44244100 |
| C | -7.53933800 | 2.82917500 | 0.67775000 |
| H | -7.92446600 | 1.88787800 | 0.27507900 |
| H | -7.44520200 | 2.71540300 | 1.76168500 |
| H | -8.28325200 | 3.60599300 | 0.48547400 |
| C | 4.49440800 | 3.32204400 | 1.39024200 |
| H | 4.55420800 | 4.24592700 | 0.80495100 |
| H | 3.68981000 | 3.46343700 | 2.12033400 |
| C | 5.81434100 | 3.08877500 | 2.11955500 |
| H | 5.76603700 | 2.18695100 | 2.73712900 |
| H | 6.63462200 | 2.96251800 | 1.40679600 |
| H | 6.06356200 | 3.92898700 | 2.77217500 |
| C | 6.84607600 | -2.01185300 | 0.81512600 |
| H | 6.37416400 | -2.19074000 | 1.78756100 |
| H | 6.83415300 | -2.96650000 | 0.27855000 |
| C | 8.28712000 | -1.55532800 | 1.02126600 |
| H | 8.78337400 | -1.38772500 | 0.06107300 |
| H | 8.32216300 | -0.61819500 | 1.58450000 |
| H | 8.86519600 | -2.30058800 | 1.57280700 |
| C | -0.93695200 | -0.89441400 | 3.01687800 |
| H | -0.31857900 | -1.34340900 | 3.80096500 |
| H | -1.35954200 | -1.72134600 | 2.43663400 |
| C | -2.06879300 | -0.08452600 | 3.64052300 |
| H | -1.67508300 | 0.75670700 | 4.21908100 |
| H | -2.72131600 | 0.32272500 | 2.86146400 |
| H | -2.67975900 | -0.69618300 | 4.30945300 |

**6**

| | | | |
|---|---|---|---|
| P | -2.28684000 | -0.08654000 | -1.36662500 |
| P | 2.23792700 | -0.42275600 | -0.53289800 |
| O | -0.05306700 | -3.69755300 | -0.74981100 |
| O | 1.75817200 | 0.87325600 | -1.31429600 |
| H | 0.96652300 | 0.42166000 | -3.00486900 |
| O | 0.30574600 | -0.02862500 | -3.55539400 |
| O | -2.72558400 | -0.64963600 | 0.05198700 |
| O | 2.29673000 | -4.00448200 | 0.73230900 |
| O | -3.42204800 | 0.16873300 | -2.28929000 |
| O | -1.19508100 | -1.12097300 | -1.82108600 |
| H | 0.12735800 | -2.80198200 | -1.07453200 |
| O | -1.40374800 | 1.21238300 | -1.07045800 |
| O | 3.79244100 | -0.17259900 | -0.36200600 |
| O | 1.86360600 | -1.65972700 | -1.25838500 |
| H | -0.56978400 | -0.75352400 | -2.54368100 |
| O | 1.68712400 | -0.31010700 | 0.94841200 |
| C | 1.48103100 | 2.15605800 | -0.68097000 |
| C | -0.42649200 | 0.94457900 | -4.31001500 |
| C | 0.46463000 | -0.97330400 | 1.38218600 |
| C | -1.38197900 | -3.76156500 | -0.24715700 |
| H | -1.53484600 | -3.04741000 | 0.56761500 |
| H | -2.11143500 | -3.56478700 | -1.03674000 |
| H | -1.53490700 | -4.77187200 | 0.13523800 |
| H | 0.22257100 | 1.40797300 | -5.05523300 |
| H | -1.23657800 | 0.41822200 | -4.81398900 |
| H | -0.84463000 | 1.71396200 | -3.65530700 |
| H | 1.04803900 | 2.75335100 | -1.48405700 |
| H | 0.72336200 | 1.99589800 | 0.08908700 |
| C | 2.72128800 | 2.81352300 | -0.11492000 |
| H | 3.13697600 | 2.19559100 | 0.68789600 |
| H | 3.48170200 | 2.89378300 | -0.89889300 |
| H | -0.14332100 | -1.22108700 | 0.50964400 |
| H | 0.76539400 | -1.89623300 | 1.88345800 |
| C | -0.28343900 | -0.04676400 | 2.31304100 |
| H | 0.41029600 | 0.35117700 | 3.06109100 |
| H | -0.66521100 | 0.80087500 | 1.73390500 |
| C | 4.62319200 | -1.20690500 | 0.23123500 |
| H | 4.67132700 | -2.04562400 | -0.46900300 |
| H | 4.15938300 | -1.53919900 | 1.16527300 |
| C | 5.99177800 | -0.61575700 | 0.48011400 |
| H | 6.38697500 | -0.22742100 | -0.46437800 |
| H | 5.89324800 | 0.22865900 | 1.17033500 |
| C | -2.02731900 | 2.51576600 | -0.96230600 |
| H | -1.21033100 | 3.22940000 | -1.07678900 |
| H | -2.72299300 | 2.64246500 | -1.79627700 |
| C | -2.72249000 | 2.70080200 | 0.37228300 |
| H | -3.52431100 | 1.96086400 | 0.48149400 |
| H | -2.00081400 | 2.52262300 | 1.17676100 |
| H | 1.42444600 | -3.95819200 | 0.29872000 |
| C | -4.11460800 | -0.81608000 | 0.43405400 |
| H | -4.12897200 | -0.63477300 | 1.50904700 |
| H | -4.72113500 | -0.05186600 | -0.05976400 |
| C | -4.60036300 | -2.20885100 | 0.08464500 |
| H | -4.00642300 | -2.94383400 | 0.63952400 |
| H | -4.42560700 | -2.38039600 | -0.98275500 |
| H | 2.85584800 | -3.47503700 | 0.15292100 |
| C | -6.08529500 | -2.39496100 | 0.40250600 |
| H | -6.67418900 | -1.69149100 | -0.19619200 |
| H | -6.38158100 | -3.39844700 | 0.08465400 |
| C | -6.41613300 | -2.21439200 | 1.88335100 |
| H | -6.26566000 | -1.18247700 | 2.21060400 |
| H | -7.45867000 | -2.47246100 | 2.08438800 |
| H | -5.78528400 | -2.85825800 | 2.50438600 |
| C | -3.31065700 | 4.10379900 | 0.50458500 |
| H | -4.03449100 | 4.27076400 | -0.30018400 |
| H | -2.51491700 | 4.84493500 | 0.37251100 |
| C | -3.98639500 | 4.30873400 | 1.85709000 |
| H | -4.79605200 | 3.58721300 | 2.00041500 |
| H | -3.27047600 | 4.17452800 | 2.67323200 |
| H | -4.41067000 | 5.31178900 | 1.94368600 |
| C | 2.37973100 | 4.19855900 | 0.43289200 |
| H | 1.96225300 | 4.81388400 | -0.37134100 |
| H | 1.59768900 | 4.10140500 | 1.19436900 |
| C | 3.59772200 | 4.89295000 | 1.03398400 |

| | | | |
|---|---|---|---|
| H | 4.01507200 | 4.30465900 | 1.85606500 |
| H | 4.38225000 | 5.02351300 | 0.28319100 |
| H | 3.33894900 | 5.87999500 | 1.42444200 |
| C | 6.95025600 | -1.65476500 | 1.05770600 |
| H | 6.52987800 | -2.06329900 | 1.98289700 |
| H | 7.04196300 | -2.49087800 | 0.35645000 |
| C | 8.32874400 | -1.06383100 | 1.33676300 |
| H | 8.77353500 | -0.66404800 | 0.42094100 |
| H | 8.26310900 | -0.24725500 | 2.06139400 |
| H | 9.00994100 | -1.81671000 | 1.74015400 |
| C | -1.43627000 | -0.77485900 | 3.00554400 |
| H | -1.02841000 | -1.48611500 | 3.73132100 |
| H | -1.99098900 | -1.36188600 | 2.26680900 |
| C | -2.38364000 | 0.19697600 | 3.70160100 |
| H | -1.85207500 | 0.79167500 | 4.45092400 |
| H | -2.82625200 | 0.89084900 | 2.97958800 |
| H | -3.19744100 | -0.32935400 | 4.20728400 |
